# HMDF-Net: a transfer learning-based heterogeneous multimodal dynamic fusion network for depression detection among inmates in correctional facilities

**DOI:** 10.3389/fpsyt.2026.1861633

**Published:** 2026-09-01

**Authors:** Zhifei Xu, Shiyun Shao, Jiagang Dong, Ziguan Wei, Yichao Zhang

**Affiliations:** 1 Zhejiang Provincial Academy of Judicial Administration, Zhejiang Police Vocational Academy, Hangzhou, China; 2 Zhejiang Provincial Engineering Research Center for Brain Cognition, Disease and Digital Medical Devices, School of Information Engineering, Hangzhou Medical College, Hangzhou, China; 3 Department of Digital Technology, Zhejiang Yuying College of Vocational Technology, Hangzhou, China

**Keywords:** correctional mental health, depression detection, dynamic attention mechanism, few-shot learning, multimodal fusion, non-cooperative environment, transfer learning

## Abstract

**Objective:**

In correctional institutions, inmates frequently suffer from mental health issues (such as depression), which can easily lead to emergencies like suicide, self-harm, and violent conflicts, posing severe challenges to security management and recidivism prevention. Traditional assessment methods have limitations such as subjectivity, lag, and low efficiency, which may result in misjudgment, omission, and delayed judgment. To address these issues, this study aims to develop an automated depression screening model suitable for the complex scenarios in correctional institutions to achieve objective, accurate, dynamic, and efficient non-contact mental health risk monitoring and early warning.

**Methods:**

A Heterogeneous Multimodal Dynamic Fusion Network (HMDF-Net) is proposed. This method first constructs a multimodal dataset for inmates and adopts a transfer learning strategy to alleviate the problem of data scarcity. HMDF-Net integrates VGG19, wav2vec 2.0, and BERT to extract visual (facial micro-expressions), audio (speech prosody), and text (dialogue semantics) features respectively. A temporal attention dynamic fusion mechanism is introduced to dynamically assign weights according to the real-time signal quality of each modal channel.

**Results:**

HMDF-Net achieves an accuracy of 87.5% and a recall rate of 100% on the test set, significantly outperforming various comparison methods. The analysis of the dynamic attention mechanism reveals an intelligent hierarchical fusion paradigm of “visual as the main (48%), text as the auxiliary (32%), and audio as the supplement (20%)”, verifying the core role of the visual modality in combating emotional concealment and the decision-making logic of the multimodal dynamic fusion model in complex environments.

**Conclusion:**

We present HMDF-Net, a cross-modal fusion network equipped with temporal attention that adjusts feature weights according to signal quality to suppress low-quality multimodal inputs. This framework offers a feasible option for scalable non-invasive mental health screening within correctional settings. Our observations suggest dataset limitations unique to this field matter more to model performance than available computing resources.

## 1 Introduction

Depression is one of the most common and dangerous causes of suicide and self-harm globally, and this issue is particularly severe in correctional facilities such as prisons and detention centers (1). The inherent closed, coercive, and high-pressure nature of the correctional environment exposes inmates to multiple stressors, including disrupted social support, restricted freedom, and an uncertain future, significantly increasing the risk of mental disorders such as depression, anxiety, and hostility (2). These mental health problems, especially depression, are often clear and dangerous precursors to emergencies such as self-harm, suicide, and violent conflicts in correctional facilities (1, 3), seriously threatening the safety and order of the facilities. In a vicious cycle, negative psychological symptoms such as depression are also important predictors of recidivism (4, 5). If not effectively corrected, they will significantly increase the risk of recidivism and pose a long-term threat to social public safety (6). Therefore, establishing a timely and accurate mental health risk early-warning system to shift from “passive response” to “active prevention” is an urgent need for the safety management of correctional facilities and the rehabilitation of offenders.

Currently, the mental health assessment of inmates in correctional facilities mainly relies on periodic scale screenings (such as SCL-90, PHQ-9, MMPI, etc.), as well as the subjective observations and experience-based judgments of correctional officers (7). These traditional methods have three key limitations: Firstly, they are static and lagging. That is, static scales cannot timely and accurately capture changes in mental states triggered by events such as family changes and interpersonal conflicts, often resulting in delayed warnings (8, 9). Secondly, there is subjective distortion. Inmates often deliberately conceal their true feelings when filling out scales in order to seek commutation of sentences or avoid being labeled as having mental abnormalities, thus generating a large number of false-negative results (10). Meanwhile, the observations of correctional officers are also limited by many factors such as personal experience, attention distribution, and interaction frequency, often leading to assessment biases and blind spots (7). Thirdly, they are inefficient and have a limited coverage. Due to the extreme shortage of professional psychological counselors in correctional facilities, it is impossible to conduct high-frequency and high-quality in-depth interviews with all inmates. Therefore, it is difficult to truly achieve regular and refined full-scale mental health monitoring. For example, in the key scenario of family visits, which have a significant impact on inmates’ emotions and psychology, the assessment of their emotional and psychological states mainly depends on officers’ manual observation of surveillance videos and listening to conversation contents. This large-scale workload can easily lead to fatigue, and the final judgments are inevitably affected by officers’ personal experience and sense of responsibility, often resulting in many problems such as misjudgment, omission of judgment, and delayed judgment (11).

Fortunately, the concurrent advancement of multimodal data ecosystems and artificial intelligence—amplified by China’s national strategies for smart correctional facilities and digital judicial governance—presents an operationalizable framework for addressing these constraints. The pervasive digital footprint within modern penitentiaries, coupled with advanced computational techniques (e.g., computer vision, natural language processing, machine learning), establishes a robust infrastructure for objective dynamic mental health surveillance (12–14). Empirically, multimodal indicators spanning vocalic, lexical, and facial expression domains have demonstrated diagnostic efficacy in automated depression detection (ADD), propelling CV/NLP-based methodologies into a burgeoning research domain (15–17). Notwithstanding, extant models predominantly leverage curated laboratory datasets (e.g., AVEC, DAIC-WOZ). Direct deployment in carceral settings confronts domain-specific implementation bottlenecks, principally manifested in:

### 1.1 The risk of overfitting in small-sample data

The performance of deep learning models usually depends on the support of large-scale labeled data. However, due to the extremely high sensitivity and difficulty of collecting correctional facility data, it is extremely difficult to construct a large-scale dataset of inmates (18). At present, although mainstream architectures such as Vision Transformer (ViT) perform excellently in general tasks (19), due to the lack of translation invariance and locality priors that convolutional neural networks (CNN) possess (20), they are prone to catastrophic overfitting when trained on correctional facility datasets with only dozens or hundreds of samples, resulting in a serious lack of model generalization ability. How to design a feature extractor that can capture subtle facial features and has good generalization ability in the absence of large-scale pre-trained data is a key challenge (21). In addition to the limited data scale, the heterogeneity of multimodal data and the deliberate emotional camouflage of the inmate population further increase the difficulty of detection (22).

### 1.2 Deliberative dissimulation in incarcerated populations

Depression manifests through multidimensional behavioral signatures. Whereas inmates may suppress affective expressions via facial control (e.g., simulated smiling), concurrent modulation of paralinguistic features (e.g., fundamental frequency jitter) and linguistic markers (e.g., negative valence lexicon) proves biomechanically challenging (23). Consequently, unimodal detection architectures demonstrate heightened vulnerability to such dissimulative behaviors (24).

### 1.3 Heterogeneity of multimodal data

The clinical manifestations of depression are heterogeneous. Therefore, diagnostic methods that go beyond single-modality data analysis are required, and complementary information from different behavioral and physiological sources needs to be integrated. Contemporary research increasingly shows that systematically integrating multimodal data— including linguistic content in text, paralinguistic features in audio, emotional cues in facial expressions, autonomic nervous system indicators in physiological signals, and behavioral markers in digital footprints—can more comprehensively reflect the underlying psychological pathological mechanisms (25). However, there are significant differences in time sampling frequency, signal quality stability, and feature representation dimensions among heterogeneous modal data such as videos, audios, and texts. The traditional feature stitching strategy can only achieve shallow-level fusion between modalities and cannot effectively model the deep semantic associations in the cross-modal interaction process (13). Therefore, for heterogeneous multi-modal data, constructing an advanced fusion architecture that can simultaneously take into account modal differences, temporal dependencies, semantic interactions, and signal quality fluctuations is the key to improving the performance of depression detection and its adaptability to different scenarios.

### 1.4 The resource-constrained correctional environment

The real correctional environment is different from a quiet psychological counseling room, often accompanied by background noise and light changes. High-performance screening models are needed to cope with the above environmental interferences. However, the internal network of the correctional settings is in a state of physical isolation, and the hardware update cycle is extremely long. An overly large end-to-end large model is difficult to run in real time on edge devices with limited performance. Therefore, when the algorithm model is actually applied to the correctional environment, it is necessary to balance the model performance and the consumption of computing resources.

In recent years, multimodal fusion methods have made significant progress in the field of depression detection. Existing research has confirmed that such complex fusion analysis methods can more accurately capture subtle depressive behavior patterns that are difficult to identify through unimodal analysis, and can output more accurate, robust, and ecologically valid assessment results, effectively filling the technical gap between model performance and actual clinical implementation.

Several representative studies have verified the technical advantages of multimodal fusion under different experimental settings: Focusing on controlled laboratory and clinical scenarios, Yang Minqiang et al. successively proposed the TSTCCA and SguiNet models, extracting spatio-temporal feature cues from facial expressions, pupil diameter, and eye-movement sequences. Both methods achieved stable detection results on the dedicated experimental datasets, verifying the clinical diagnostic potential of multimodal biomarkers such as facial expressions, pupil diameter, and eye-movement (26, 27). Breaking free from the limitations of the fixed laboratory environment, Tao Yongfeng et al. constructed an efficient, low-covariance multimodal integrated spatio-temporal transformer framework-DepMSTAT, which aims to use acoustic and visual features in social media data to identify depressive states. This framework consists of four modules: a data pre-processing module, a label generation module, a spatio-temporal attention transformer (Stat) module, and a depression classification module. To effectively capture the spatial and temporal correlations of multimodal social media depression data, a plug-and-play STAT module was proposed. This module can extract unimodal spatio-temporal features and fuse unimodal information, playing a key role in analyzing the acoustic and visual features in social media data (28).

Existing studies have shown that the multi-modal depression detection paradigm has gradually evolved from the early shallow feature splicing and fusion to the high-order technical route focusing on spatio-temporal dependency modeling, attention mechanism enhancement, redundant feature suppression, and robust cross-modal interaction. Although existing methods have achieved considerable performance in their respective controlled experimental environments, and it has been verified that the eye dynamic behavior features induced by emotional stimuli can provide a strong discriminative basis for depression recognition, their performance highly depends on the standardized experimental stimulus paradigm, high-fidelity eye movement video collection conditions, and the additional semantic pseudo-label supervision constraints introduced. Therefore, it is difficult to directly migrate them to the uncontrolled complex environment in detention facilities, where there are frequent eye occlusions, significant head movement interferences, low cooperation of subjects, and deliberate emotional camouflage, resulting in continuous fluctuations in modal quality.

Specifically, the current relevant cutting-edge research mainly focuses on four technical paths: intra-modal temporal modeling, cross-modal attention interaction, perceptual reliability fusion, and modal missing handling. Although these approaches can improve the complementary utilization efficiency of multi-source information to some extent, they still have significant limitations in the non-cooperative complex scenarios of correctional facilities.

The first type is the method based on intra-modal temporal attention. By introducing temporal modeling mechanisms such as self-attention or bidirectional long-short-term memory (Bi-LSTM) networks into the uni-modal encoder, long-range temporal dependencies of facial expressions, speech rhythms, or text semantics are captured (29). However, these methods only optimize temporal features within the modality and do not solve the problem of dynamic fluctuations in signal quality differences between channels from the perspective of cross-modal interaction. The second type is the method based on inter-modal fusion attention. For example, Multimodal Transformer (MulT) achieves temporal alignment and semantic interaction between modalities through cross-modal attention. However, these methods usually focus more on the modeling of modal relationships at the global level, that is, assigning a single fixed weight to each modal stream. The implicit assumption is that modal reliability is uniformly distributed over the entire time axis, and they have insufficient response to short-term and uneven local signal quality changes caused by factors such as personnel movement, occlusion, and instantaneous device noise in detention facilities, which can easily lead to the wrong inclusion of noise information from contaminated segments in the fusion or the suppression of high-quality discriminative information from clear segments (30).The third type is a newly emerging reliability-aware fusion method in recent years, including methods such as modality-attention mechanisms, quality-aware fusion, and uncertainty-based fusion. This type of method attempts to dynamically evaluate the overall signal quality of the modality (31). However, most of the existing schemes rely on explicit signal quality annotations or Bayesian uncertainty estimation. The former is difficult to obtain reliable training signals in non-cooperative scenarios where detainees generally have a defensive mentality, while the latter not only has high computational costs but also has unreliable uncertainty estimation results under the condition of small-sample data unique to this study. The fourth type is the handling method for the problem of modal missingness. These techniques are mainly designed for extreme scenarios where a certain modality is completely unavailable and are difficult to adapt to the actual characteristics of “the modality exists but the quality of local segments fluctuates continuously” in correctional facilities.

In response to the limitations of existing multimodal fusion schemes in the implementation within the correctional scenarios, this research proposes a lightweight dynamic fusion framework called HMDF-Net. Different from previous fusion strategies that rely on static global weights or can only handle complete modality loss, HMDF-Net achieves fine-grained dynamic weighting at the time-step level. It can perform modality reliability assessment at each time node, accurately filter out noisy and low-quality segments, and retain effective features with high distinguishability. It dynamically adjusts the contribution proportion of each modality in the final fusion result based on the real-time feature quality of each modality, thereby adapting to the frequent local signal fluctuation characteristics that occur during the psychological screening of inmates. HMDF-Net provides a targeted solution for robust depression detection in complex correctional scenarios, and has potential application value in prison and detention center scenarios where edge computing resources are limited and the collection environment is controllable but not ideal (for example, there is still background noise in the standardized interview room).

In summary, the main contributions of this paper include three aspects:

#### 1.4.1 Specific-domain dataset

We provide a multi-modal depression assessment dataset from correctional institutions, which contains the corresponding multi-modal audio-visual feedback data of 76 inmates and the data of three types of psychological assessment scales such as compulsion, depression, and anxiety. This fills a key gap in the research on the mental health of inmates.

#### 1.4.2 Methodological framework

We propose HMDF-Net, a lightweight, time-step-level dynamic weighting mechanism specifically designed for small-sample, synchronously aligned multi-modal sequences. It further confirms the feasibility and potential of multi-modal fusion with reliability perception.

#### 1.4.3 Empirical insights

Through a systematic ablation study, we prove that compared with the static fusion baseline and other simple modality-level weighting methods, dynamic modality weighting fusion can improve the performance of depression detection, which will promote future research on robust multi-modal learning under real-world interference.

## 2 Materials and methods

### 2.1 Dataset construction

This study is a real-world cross-sectional retrospective study. The research team collaborated with the judicial correction management department of a province in eastern China. From May to July 2023, they collected multi-modal psychological data from a total of 76 inmates in three different correctional institutions in this region. The data mainly included three types of audio-visual data, namely picture descriptions, psychological interviews, and read texts, as well as the corresponding data from three types of psychological assessment scales for compulsion, depression, and anxiety. The research team used the Oddball experimental paradigm to provide standardized emotional stimuli and collected the corresponding multi-modal feedback data to identify the inmates’ emotional and psychological states.

The research team established strict inclusion and exclusion criteria to ensure the reliability and validity of the research dataset. The inclusion criteria are as follows: (1) Aged 18 years or older; (2) Voluntarily participating and fully understanding the research purpose and procedures; (3) Having sufficient verbal communication ability and cognitive function to complete all assessment tasks; (4) Having a stable physical condition to support the completion of the entire data collection process. Potential participants meeting any of the following criteria will be excluded: (1) Being diagnosed with severe mental disorders, such as schizophrenia; (2) Having a recent history of drug abuse; (3) Having significant cognitive impairment (e.g., dementia); (4) Suffering acute from or uncontrolled medical conditions that interfere with participation in the assessment.

This study design was approved by the Institutional Review Board of Zhejiang Police Vocational Academy (Number: 21G010006) on May 12, 2021, and the entire process strictly adhered to the Declaration of Helsinki and relevant national judicial research norms. Researchers accessed and used data solely for analysis purposes, and all data of the subjects under study (including patients) were anonymized. Before data collection, all participants were informed of the study’s purpose, procedures, potential risks, and the right to withdraw, and they signed written informed consent forms. Participation in this study followed the principle of voluntariness. Refusal to participate or withdrawal during the study would not affect a participant’s legal status, parole eligibility, or imprisonment conditions.

The diagnosis of depression among inmates is made by two psychiatrists who are board-certified and have over five years of clinical experience, in accordance with the “Chinese Classification and Diagnostic Criteria of Mental Disorders (Third Edition)” (CCMD-3). This standard is consistent with the diagnostic criteria of the “International Classification of Diseases (Tenth Edition)” (ICD-10).

### 2.2 Oddball experimental paradigm design

To overcome the defensive psychology of inmates in conventional assessments, this study introduced the Oddball experimental psychology paradigm, a commonly used experimental design method in psychological and cognitive neuroscience research, which is specifically used to study the brain’s response mechanism to accidentally occurring deviant stimuli (32). More importantly, this paradigm can induce the subjects’ true psychological feedback in an involuntary state through emotional stimuli with a fixed probability. Therefore, the researchers designed an experimental paradigm for depressive symptoms with three types of stimulus tasks (picture description, interview, and text reading) and three types of emotional valences (positive, neutral, and negative) to induce behavioral differences between healthy individuals and those with depressive symptoms.

This experiment consists of three distinct and sequential task segments, with a total duration of approximately 15 minutes (each participant completes 16 segments, taking about 15 minutes in total, and the overall data collection for the entire sample lasts 1140 minutes, equivalent to 19 hours).

#### 2.2.1 Picture description (emotion induction)

First, select three facial expression pictures (happy, sad, and calm) from the Chinese Facial Affective Picture System (CFAPS) and the Chinese Affective Picture System (CAPS). The viewing of the pictures by the participants and the recording start simultaneously and continue until the description ends. Ensure that the participants’ facial expressions and voices are completely recorded throughout the induction process.

#### 2.2.2 Semi-structured interviews (stress response)

The participants are required to answer 10 specific questions (as shown in **Table 1**), including 2 questions with positive valence, 3 with neutral valence, and 5 with negative valence. All the questions are derived from the integration and modification of assessment scales or questionnaires such as SCL-90, PHQ-9, HDRS, BDI, and the DSM manual (33–35), covering future plans, past failures, and interpersonal conflicts to induce in-depth emotional exposure. The statement of questions and the recording start simultaneously, and facial expressions and voices are collected at the same time.

**Table 1:** Interview transcripts.

Order number	Interview text
1	Please share a memory you cherish most and describe the scene in broad terms.
2	Please state your plan for the next three years.
3	How is your recent physical condition? How is your energy level?
4	How have you been feeling lately? For example, are you feeling lonely, depressed, anxious, or easily stressed?
5	Do you often feel nervous or easily nervous?
6	Please share what made you feel like a failure, including your thoughts and actions at the time.
7	Do you have the habit of checking things repeatedly when you do things?
8	Do you often feel uneasy in your mind? Why?
9	How have you been with your family recently?
10	Have you had any conflicts with your companions or friends recently? If so, why?

#### 2.2.3 Text reading (baseline calibration)

Have the subjects read three texts with different emotional valences (positive, neutral, and negative) selected from the Chinese emotional ontology corpus. The reading and recording start simultaneously, and the voice and facial expressions are continuously and objectively recorded throughout the process, with a focus on subtle prosodic changes. When labeling, the “double-verification” method is adopted: that is, the scores of self-assessment scales such as PHQ-9, GAD-7, and Y-BOCS, as well as the results of psychological interviews, are combined to ensure accurate classification.

### 2.3 Data collection and diagnostic labeling

**Figure 1:**
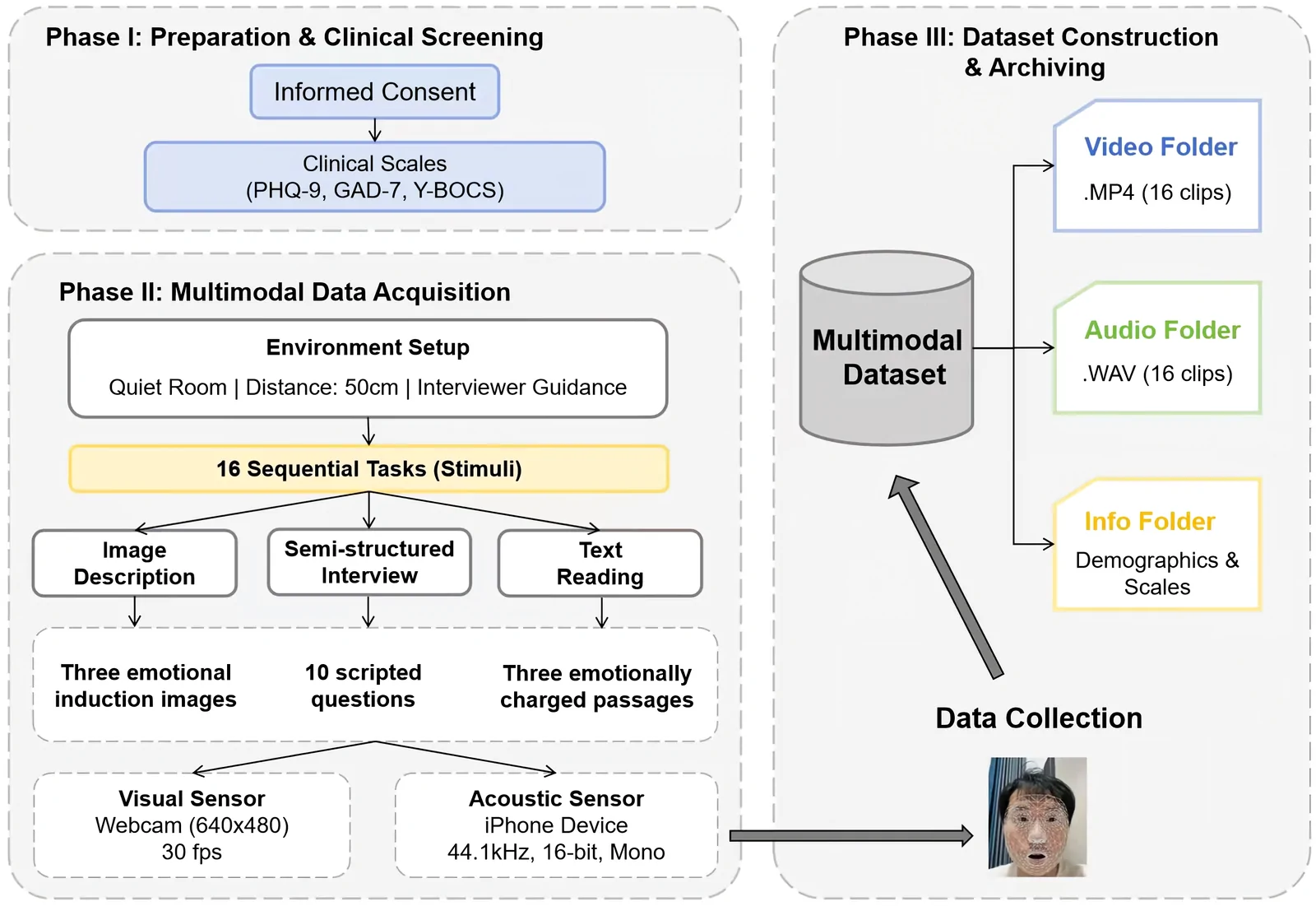
Data acquisition pipeline for multimodal psychological assessment.

Data collection was conducted in a quiet and dedicated psychological interview room, and video recordings were made. Subsequently, the participants filled out the self-assessment scales of PHQ-9, GAD-7, and Y-BOCS for subsequent annotation purposes (36, 37). More importantly, the “double-verification” method was adopted for label annotation: the scores of the PHQ-9, GAD-7, and Y-BOCS self-assessment scales were combined with the interview results of the psychological counselors for strict classification. During the recording, the participants sat 50 centimeters away from the computer screen, and the recording device was placed at a standard distance from the mouth. Three tasks (picture description, semi-structured interview, and text reading) were presented on the screen in sequence, and the participants completed the tasks according to the screen prompts. The recorded audio was captured using an iPhone device, with a sampling rate of 44.1 kHz, 16-bit quantization, and a dual-channel format. Due to the symptoms of depressed patients such as low mood, slow speech rate, and inability to concentrate, the interviewers needed to actively and patiently provide appropriate guidance throughout the recording process. The data collection process is shown in **Figure 1**.

Depression diagnosis is clearly based on the Chinese Classification and Diagnostic Criteria of Mental Disorders (3rd Edition, CCMD-3). That is, patients must have one of the core symptoms of depressed mood or loss of interest and pleasure in the past two weeks, and be accompanied by at least 3 additional symptoms, which cause significant impairment in social interaction, occupational activities, or other important functional areas. Moreover, all symptoms cannot be solely attributed to substance use or other medical diseases (36).

### 2.4 Data preprocessing

**Figure 2:**
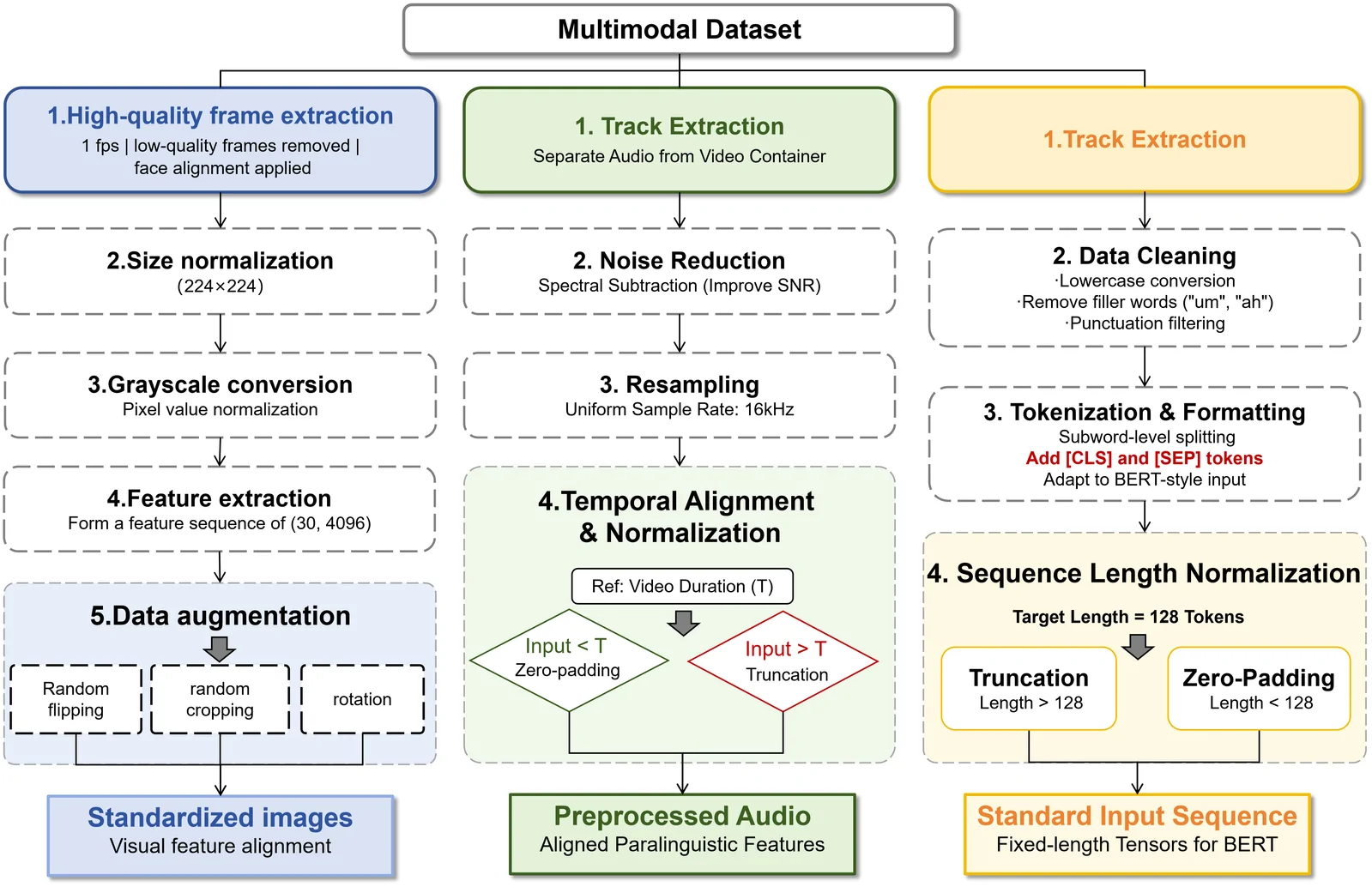
Overview of multimodal data preprocessing pipeline.

Data preprocessing is a fundamental step in constructing multimodal deep learning models. To eliminate the biases among heterogeneous data and ensure the consistency of input dimensions, this study has developed a standardized processing pipeline for the three modalities of video, audio, and text, covering numerous aspects such as data cleaning, spatio-temporal alignment, and normalization (as shown in **Figure 2**).

#### 2.4.1 Video modal preprocessing

To obtain standardized visual inputs from the original interview videos, we first segmented the original long-form videos into independent clips based on the timestamps of the interview questions. Then, we extracted a sequence of static images at a frequency of 1Hz (i.e., one frame per second) as the basic units for video analysis. Subsequently, we carried out strict quality control and standardization processes on the extracted frame sequences. We removed low-quality frames where the face could not be recognized due to motion blur or severe occlusion to ensure the effectiveness of the input data. Face detection and key point alignment were performed using the OpenFace tool, and the region of interest (ROI) of the face was cropped. Subsequently, the sizes of all images were uniformly adjusted to 224×224 pixels to meet the input specifications of the convolutional neural network. Since color information contributes limitedly to facial emotion recognition and is easily affected by ambient light, all images were converted to grayscale, and the pixel values were normalized. This not only accelerated the model convergence speed but also enhanced the robustness against illumination changes. During the sample construction phase, we packaged consecutive 30-frame images into a standard input sample sequence to fully preserve the temporal evolution features of facial expressions. For each video clip containing 30 frames, we input the frames one by one into VGG19 and extracted the 4096-dimensional feature vectors of each frame at the fc7 layer. Finally, we obtained a feature sequence with a shape of (30, 4096), which served as an ideal input for the subsequent Bi-LSTM. In addition, this paper introduced various geometric transformation techniques such as random flipping, random cropping, and rotation to augment the training data. This effectively expanded the sample diversity and avoided model overfitting.

#### 2.4.2 Audio modal preprocessing

The focus of audio modal preprocessing lies in noise reduction and temporal alignment, aiming to accurately extract paralinguistic features from speech. The processing procedure is as follows: First, the audio track is separated from the video container, and Spectral Subtraction is applied to remove background environmental noise to improve the signal-to-noise ratio. Subsequently, the audio sampling rate is uniformly resampled to 16 kHz to strictly meet the input specifications of the subsequent acoustic feature extraction model. To ensure the temporal consistency of multimodal data, the audio stream is precisely cropped and aligned according to the duration of the corresponding video segments. For segments with insufficient length, a zero-padding strategy is adopted for completion, while for overly long segments, truncation processing is carried out, thus ensuring a clear and strict correspondence between each audio input and the video sequence on the time axis.

#### 2.4.3 Text modal preprocessing

The text data is derived from the transcription of interview voices. Its preprocessing aims to maximize the retention of semantic information and eliminate irrelevant noise. The processing flow is as follows: First, perform standardized cleaning on the original transcribed text. Specific operations include converting all English characters to lowercase, removing meaningless modal particles (such as “um”, “ah”), and filtering non-semantic punctuation marks. Subsequently, use the Subword (sub-word) granularity segmentation method to perform Tokenization on the text, and add the [CLS] token at the beginning of the sentence and the [SEP] token at the end of the sentence to adapt to the input format of the pre-trained language model. To meet the requirements of batch training, we set the maximum sequence length to 128 Tokens. Texts exceeding this length are truncated, and the insufficient parts are padded, ultimately generating a standard input sequence of a fixed length.

### 2.5 Model framework overview

**Figure 3:**
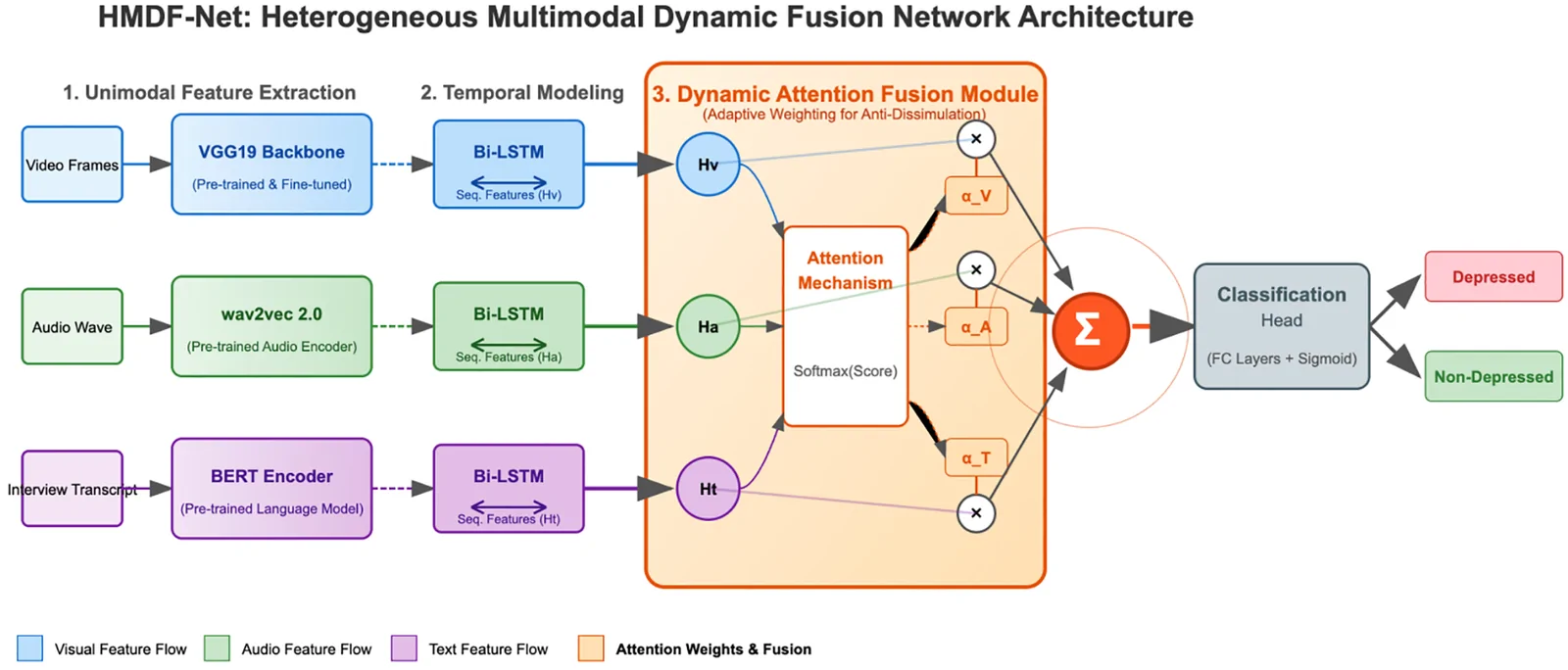
Schematic of HMDF-Net architecture.

In response to the severe challenges such as data scarcity and serious noise interference in the non-cooperative environment of detention facilities, this paper proposes a Heterogeneous Multimodal Dynamic Fusion Network (HMDF-Net). This framework combines the transfer learning strategy and the temporal attention mechanism, aiming to achieve robust depression screening in detention facilities by deeply mining the complementary information among three modalities, namely video, audio, and text (as shown in **Figure 3**). HMDF-Net consists of three cascaded computational stages: heterogeneous feature extraction, temporal dependency modeling, and dynamic attention fusion.

Firstly, in the feature extraction stage, the model designs three independent parallel branches for the physical characteristics of multimodal data. For the video stream, we use the VGG19 backbone network pre-trained on a large-scale dataset as the feature extractor. Then, we freeze the shallow parameters of this network to retain its ability to extract general visual features (such as edges and textures). At the same time, we fine-tune the deep-layer network and the classification head using small-sample data to achieve the learning of high-level semantic features for specific tasks, so as to accurately capture micro-expressions and effectively prevent the model from overfitting. The audio stream relies on the wav2vec2.0 model, aiming to lock in the paralinguistic acoustic representations contained in the speech. The text stream is deeply modeled through the BERT encoder to mine the underlying semantic logical structure of the text.

Secondly, in the stage of temporal dependency modeling, the model first uses the Bi-LSTM to encode the frame-level or word-level feature sequences extracted from VGG19, wav2vec 2.0, and BERT respectively. Through its gating mechanism, Bi-LSTM can capture the long-term temporal dependency relationships of the features within each modality, and transform the discrete feature points into temporal hidden vectors (H_v_, H_a_, H_t_) containing contextual semantics. This step is an implicit temporal attention process, which determines how the model understands the development context of events within each modality and provides a feature representation rich in temporal context information for the subsequent cross-modal fusion.

Finally, the Dynamic Attention Fusion Module is responsible for the integration of cross-modal information. After obtaining the temporal feature representations of each modality, the HMDF-Net introduces the Dynamic Attention Fusion Module. Different from traditional static concatenation or fixed-weight fusion, the core of this fusion strategy is a learnable parameterized attention mechanism. Its dynamic nature is manifested in the following: at each time step t, instead of using fixed weights, this mechanism calculates an attention energy score $e_{m}^{t}$ in real-time based on the hidden states $h_{m}^{t}$ of each modality at the current moment, and normalizes it through the Softmax function to obtain the fusion weights $\alpha_{m}^{t}$ of each modality at this moment. That is to say, this strategy can adaptively calculate the confidence weights (*α*_v_, *α*_a_, *α*_t_) according to the real-time quality and discriminative contribution of each modality at the current time step. Through the weighted summation operation, the model can automatically suppress the modalities interfered by high noise or camouflage behaviors and enhance the expression of complementary features. Finally, the generated fusion vector is output through the fully-connected classification head to obtain the depression risk prediction results.

### 2.6 Heterogeneous feature encoding based on transfer learning

To address the challenges of limited sample sizes and data accessibility in the screening of depression in correctional settings, training deep neural networks with random weight initialization frequently yields local optima or overfitting. This study therefore adopts a transfer learning strategy via parameter fine-tuning. Specifically, this approach leverages generalized feature representations pre-trained on large-scale source domains (e.g., ImageNet, LibriSpeech, Wikipedia) and adapts these representations to the inmates via differential fine-tuning, balancing the retention of general features with the extraction of domain-specific affective representations.

#### 2.6.1 Visual feature encoding

In the visual modality, architectures such as Vision Transformer (ViT) often face the problem of unstable convergence in small-sample training scenarios due to the lack of inductive bias. Therefore, this study adopts the VGG19 architecture with excellent transfer learning ability as the visual backbone network (38). The low-level convolutional kernels of VGG19 pre-trained on the ImageNet dataset can capture general edge and texture features with strong generalization ability and suitability for cross-domain tasks, while the high-level convolutional kernels can perform high-level semantic abstraction, which is highly suitable for the task requirements of facial micro-expression analysis.

To adapt VGG19 for depression-related micro-expression detection, a layer-wise freezing strategy is applied without excessive parameter tuning. The first 10 shallow convolutional layers are frozen, as they encode basic geometric visual features that are relatively invariant to domain shifts. Only the deep convolutional layers and fully connected layers are fine-tuned. This scheme reduces the number of trainable parameters by approximately 40%, guiding the model to concentrate on learning depression-specific facial micro-expression semantics (e.g., furrowed glabellar lines, drooping mouth corners) rather than re-learning low-level image features.

For each frame v_t_ in the input video sequence, we extract the output of the fully connected layer fc7 as the high-dimensional visual feature vector $x_{v}^{t}$, as shown in Equation 1.

(1) $$x_{v}^{t}=\sigma \left(W_{fc7}\cdot {ℱ}_{conv}\left(v_{t};\theta_{frozen},\theta_{fine}\right)+b_{fc7}\right)\in {\mathbb{R}}^{4096}$$

where *θ_frozen_* and *θ_fine_* denote the frozen and fine-tuned network parameters, respectively, and *σ* denotes the ReLU activation function. The resulting visual feature dimension is *d*_v_ = 4096.

#### 2.6.2 Acoustic feature encoding

The recording environment in correctional facilities often contains a large amount of echo and background noise. In such a noisy environment, traditional methods of extracting voice features, such as MFCC, are easily interfered with, and their effectiveness is greatly reduced. To address this pain point, this study introduces the wav2vec 2.0 model, which can perform “Contrastive Learning” in a large amount of unlabeled general speech data in advance. This is like allowing the model to experience various complex sound scenes in advance and develop strong anti-noise and discrimination abilities. Therefore, it can accurately capture implicit paralinguistic cues such as the speaker’s tone and emotion in a cluttered background (39). We transfer the self-supervised knowledge obtained through this pre-training to the correctional environments. By fine-tuning the downstream tasks on limited interview recording data, the model can quickly adapt to the extraction of paralinguistic cues in a noisy environment.

For each aligned audio segment *A*_t_, wav2vec 2.0 encodes the input through a multi-layer convolutional feature encoder and a Transformer context network. Then, the outputs of the context encoder are aggregated via average pooling to derive the global acoustic embedding $x_{a}^{t}$ at each time step, as shown in Equation 2:

(2) $$x_{a}^{t}=AvgPool\left({ℱ}_{wav2vec}\left(A_{t};\theta_{a}\right)\right)\in {\mathbb{R}}^{768}$$

the resulting feature dimension is *d_a_* = 768. This embedding effectively encodes characteristic depression-related acoustic patterns, including reduced pitch and slowed speech rate.

#### 2.6.3 Text feature encoding

This study employs the BERT model to extract text features to represent the language logic and negative cognitive patterns of inmates during stress interviews (40). As an architecture based on the bidirectional Transformer, the BERT model can effectively capture the complex context-semantic dependencies between lexical items.

First, the BERT model is pre-trained on a large-scale corpus to encode rich syntactic structures and general semantic knowledge. Then, it is fine-tuned on the transcripts of inmates’ interviews to adjust the distribution mapping of the general semantic space to the specific semantic space of psychological assessment in correctional facilities.

Specifically, we input the transcribed text sequence *S_t_* into the BERT-base-Chinese model and use its bidirectional attention mechanism to encode the context dependencies of the input sequence. To obtain the semantic representation of the entire sentence, we extract the hidden state vector corresponding to the special [CLS] token, as shown in Equation 3:

(3) $$x_{t}^{t}=BERT{\left(S_{t};\theta_{t}\right)}_{\left[CLS\right]}\in {\mathbb{R}}^{768}$$

The output dimension *d_t_* = 768. This vector is rich in deep semantic information about the sense of despair, helplessness, and negative words, providing reliable textual clues for subsequent multimodal fusion.

### 2.7 Dynamic fusion mechanism

Given the complex challenges in correctional settings, including high ambient noise, visual occlusion interference, and emotional camouflage, unimodal data or static fusion rules are often insufficient to comprehensively characterize an individual’s mental health status. In contrast, the dynamic fusion mechanism can perceive variations in external conditions in real time and automatically adjust the weight distribution of different modalities. This capability ensures that the system maintains efficient and stable performance across diverse scenarios, facilitating optimal decision-making and response. The core of this mechanism lies in two key principles: dynamic adaptation and multimodal collaboration (41).To this end, this study proposes a dynamic fusion strategy that organically combines intra-modal temporal dependencies with inter-modal dynamic attention weighting.

#### 2.7.1 Intra-modal temporal modeling

Depressive emotional expressions exhibit pronounced temporal continuity, such as persistent emotional blunting or prolonged low mood. Direct fusion of frame-level features discards critical temporal context. To address this limitation, multimodal features are processed using a Bi-LSTM network to capture temporal dependencies (42), formulated as shown in Equations 4–6:

(4) $${\vec{h}}_{m}^{t}=LSTM\left(x_{m}^{t},{\vec{h}}_{m}^{t-1}\right)$$

(5) $${\overset{\leftarrow }{h}}_{m}^{t}=LSTM\left(x_{m}^{t},{\overset{\leftarrow }{h}}_{m}^{t+1}\right)$$

(6) $$h_{m}^{t}=\left[{\vec{h}}_{m}^{t};{\overset{\leftarrow }{h}}_{m}^{t}\right]\in {\mathbb{R}}^{2k}$$

where m∈{v,a,t} denotes the modality index, and *k* denotes the dimension of the LSTM hidden state. This architecture exploits both past and future contextual information to produce latent states $h_{m}^{t}$ with rich temporal semantics.

#### 2.7.2 Inter-modal attention weighting

The detailed computational pipeline of our learnable parameterized dynamic attention fusion module is elaborated as follows, and its complete structural flow is presented in **Supplementary Figure 1**. In practical correctional scenarios, single-modal detection systems frequently suffer from severe performance degradation under environmental disturbances such as facial occlusion and background noise. The energy fraction $\;e_{m}^{t}$ of each modality at time step *t* is first calculated as:

In practical correctional scenarios, single-modal detection systems often suffer a significant decline in performance due to environmental interference factors such as facial occlusion and background noise. To enhance the robustness of the system, we designed a Dynamic Attention Mechanism, aiming to automatically learn the “confidence” of each modality at different times. First, calculate the energy score $e_{m}^{t}$ of each modality at time *t*, as shown in Equation 7:

(7) $$e_{m}^{t}=v_{attn}^{T}\tanh(W_{attn}h_{m}^{t}+b_{attn})$$

where *W_attn_* and *b_attn_* denote the learnable weight matrix and bias term, respectively, and *v_attn_* represents the attention context vector. Then, the Softmax function is used to normalize the energy scores to obtain the attention weights $\alpha_{m}^{t}$ t of each modality, as shown in Equation 8:

(8) $$\alpha_{m}^{t}=\frac{\exp(e_{m}^{t})}{\sum_{j\in \left\{v,a,t\right\}}\exp(e_{j}^{t})}$$

Finally, the multimodal fused representation *Z^t^* is obtained as the weighted sum of the hidden states across all modalities as shown in Equation 9:

(9) $$Z^{t}=\sum_{m\in \left\{v,a,t\right\}}\alpha_{m}^{t}h_{m}^{t}$$

This mechanism enables the model to adaptively prioritize high-quality modalities and attenuate the influence of noisy ones.

#### 2.7.3 Focal loss for model optimization

Owing to the severe class imbalance inherent in depression screening tasks, where non-depressed cases far outnumber their depressed counterparts, standard cross-entropy loss inevitably biases the model toward the majority class. To alleviate this issue and guide the model to focus on hard, borderline depressive samples, Focal Loss is employed as the optimization objective (Equations 10):

(10) $$L_{FL}\left(p_{t}\right)=-\alpha {\left(1-p_{t}\right)}^{\gamma }\log(p_{t})$$

In this formulation, p_t_ represents the predicted probability of the model for the true category. α is the balancing factor, which is used to adjust the weight ratio of positive and negative samples. γ is the Focusing Parameter. When γ > 0, the model reduces the weight contribution of easily classifiable samples (where p_t_ is close to 1), thereby concentrating the focus of gradient updates on difficult-to-classify samples. After hyperparameter search on the validation set, this study sets *α* = 0.25 and γ = 2.0.

### 2.8 Model evaluation

To comprehensively evaluate the performance of the model in the task of depression screening in correctional facilities, this study adopted accuracy, precision, recall, F1-score, and the area under the receiver operating characteristic curve (AUC) as evaluation metrics. Considering the particularity of the psychological screening scenario in correctional facilities, recall was prioritized as the primary metric to minimize missed detections and mitigate potential safety hazards. This study used calibration curves and decision curve analysis (DCA) to validate the predictive reliability and clinical applicability of the model. The calibration curve quantifies the consistency between the predicted depression probability and the actual risk labels, and DCA evaluates the clinical net benefit at different threshold probabilities.

Five controlled ablation experiments are conducted to validate the effectiveness of core components in HMDF-Net, including training strategy, multimodal fusion mechanism, visual backbone, text encoder and audio encoder. All model variants are tested under identical experimental configurations with the aforementioned uniform metrics for fair comparison.

Furthermore, the t-SNE algorithm is employed to project high-dimensional features from the penultimate fully connected layer onto a 2D plane for feature distribution visualization. Spatio-temporal visualization of multimodal attention weights is also performed to dissect the model’s dynamic fusion process and intrinsic decision-making mechanism.

### 2.9 Risk stratification strategy

In actual clinical scenarios, the graded assessment of the severity of depression is crucial for formulating personalized intervention plans and optimizing the allocation of mental health management resources in correctional institutions. This study adopted an exploratory approach of “binary main model+probability binning and stratification” for the graded assessment of depression severity instead of directly training a five-level classification model, mainly based on the following considerations:

Limited sample size makes it difficult to stably train a multi-level classification model. Data acquisition in correctional institutions is subject to multiple constraints such as ethics, safety, and management. This study only included 76 subjects, among which 19 were in the depression group. If a five-level classification were to be directly conducted, the sample sizes of each severity level would be extremely sparse, with only a few people in the most severe level. This would easily lead to unstable model training and unreliable performance evaluation.

Binary classification diagnosis is the clinical gold standard. The diagnostic labels in this study are based on the CCMD-3 standard, and a binary diagnosis (depression/non-depression) was made by psychiatrists, which is the standard way of clinical mental illness diagnosis. The PHQ-9 scale score serves as an auxiliary assessment tool to quantify the severity of symptoms rather than replacing the clinical diagnosis.

The binary classification+binning approach is more robust and practical. Under the current data conditions, the binary classification model can ensure training stability and result reliability. On this basis, stratifying the severity through probability binning can provide clinically valuable severity information without changing the main model architecture, taking into account both the accuracy of the assessment and the practicality of the application.

This study referred to the five-level classification standard of the commonly used PHQ-9 depression scale in clinical practice and divided the continuous depression probabilities output by the model into five clinically meaningful risk levels at equal intervals (as shown in **Table 2**):

**Table 2:** Risk level classification.

Risk level	Probability range	Corresponding PHQ-9 score reference	Clinical significance
No/Extremely mild risk	0.0-0.2	0–4 points	Routine monitoring
Mild risk	0.2-0.4	5–9 points	Psychological counseling, regular follow-up
Moderate risk	0.4-0.6	10–14 points	Psychological intervention+ Drug evaluation
Severe risk	0.6-0.8	15–19 points	Drug treatment+Close monitoring
Extremely severe/Emergency risk	0.8-1.0	20–27 points	Emergency intervention, Suicide risk assessment

It should be noted that this naming is only used for the reference division of probability intervals to facilitate clinical interpretation; the real diagnostic labels in this study are still the binary classifications (depressed/non-depressed) based on the CCMD-3 criteria.

## 3 Results

### 3.1 Study subjects and experimental environment

#### 3.1.1 General information description

This study involved 76 inmates as participants (as shown in **Table 3**). During the research, three types of video and audio data were collected from each subject, including image description tasks, psychological interviews, and reading aloud tasks. Corresponding psychological scale data were also obtained, covering obsessive-compulsive symptoms, depressive symptoms, and anxiety symptoms. Each participant provided 16 data segments with a total duration of approximately 15 minutes, resulting in an overall data collection time of 1140 minutes, i.e., 19 hours.

**Table 3:** Description of general information of inmates.

Characteristics	n (%)or mean	Characteristics	n (%)or mean
Age	39.4	criminal record	
Gender		Yes	5 (6.58%)
Male	49 (64.47%)	No	71 (93.42%)
Female	27 (35.53%)	Length of incarceration	
Degree of education		1 to 3 years	48 (63.16%)
Illiteracy	1 (1.32%)	3 to 5 years	19 (25%)
Primary school	7 (9.21%)	More than 5 years	9 (11.84%)
Junior high school	4 (5.26%)	Recidivism	
Senior high school	9 (11.84%)	Yes	21 (27.63%)
Vocational school	2 (2.63%)	No	55 (72.37%)
Junior college school	27 (35.53%)	Occupation before arrest	
University	23 (30.26%)	Private entrepreneurs and individual workers	9 (11.84%)
Other	3 (3.95%)	Employee	22 (28.95%)
Household registration		Public organization staff	22 (28.95%)
Cities	24 (31.58%)	Unemployed	12 (15.79%)
Rural areas	52 (68.42%)	Retirees	1 (1.32%)
		Other	10 (13.16%)

#### 3.1.2 Study subjects and data partition

This study employed a small-scale dataset comprising 76 subjects of correctional facilities. The dataset was split such that 10% (8 subjects) were reserved as a held-out internal validation subset, which was fully isolated throughout model training and hyperparameter tuning and exclusively used for final evaluation. The remaining 90% (68 subjects) constituted the development set for model training and internal validation.

#### 3.1.3 Experimental environment and training strategies

The experiment was carried out on Ubuntu 20. 04 LTS with PyTorch 1. 13. 1, and the underlying hardware was an Intel Core i7-12700K CPU together with an NVIDIA GeForce RTX 4070Ti GPU(12GB video memory), which made computation much more efficient.

The evaluation used five-fold cross-validation on the development set, and averaging the results over the five tests helped reduce random bias. We used segment-level samples for model training and inference. Each participant yielded 16 multimodal interview segments, generating 1,216 samples from 76 inmates (19 depressed, 57 non-depressed). Eight participants (3 depressed, 5 non-depressed, 128 segments) were reserved as the held-out internal validation subset, while the other 68 subjects formed the development set for 5-fold cross-validation. All splits follow participant-level grouping: all clips and augmented data of one subject remain in the same fold, and augmentation is only executed inside each fold’s training subset to prevent data leakage. Fold-wise participant and segment statistics are summarized in **Supplementary Table 1**, which reports the participant-level depressed/non-depressed class distribution for every fold.

More importantly, the training process was clearly divided into two well-defined steps: First, fine-tune the feature extraction backbone models (deep network of VGG19, BERT, wav2vec2.0) with a low learning rate of 1*e* − 5. Subsequently, freeze the backbone network and train the back-end fusion network (Bi-LSTM and attention module) with an initial learning rate of 1*e* − 4. The AdamW optimizer is adopted, with the weight decay set to 1*e* − 3 and the Batch Size set to 16. An early stopping mechanism is introduced. When the Loss on the validation set no longer decreases within 10 Epochs, the training automatically is terminated. This simple rule prevents the model from over-memorizing the training data.

#### 3.1.4 LLM baseline evaluation settings

Because the versions of Qwen2.5-7B-Instruct and GLM-4-9B used in this study do not directly accept raw audio-visual streams, we defined a standardized evaluation procedure for these LLM baselines. Specifically, pre-extracted structured visual features, including facial kinematic indicators obtained using OpenFace, and structured acoustic features, including speech rate, pitch deviation, and pause ratio, were converted into structured textual summaries using rule-based templates. These summaries were then concatenated with the interview transcripts as model inputs.

During inference, six non-overlapping few-shot examples were selected separately for each cross-validation fold. All examples were sampled only from the training subset of the corresponding fold and included three depressed participants and three non-depressed participants. No participant from the corresponding test fold was included in the few-shot examples. Fixed decoding settings were used during inference: temperature = 0, top_p = 1.0, and max_new_tokens = 16. The models were constrained to output only two labels: “Depressed” for the positive class and “Non-depressed” for the negative class.

If the generated output did not conform to the predefined format, inference was repeated once. If the output remained malformed after this retry, it was recorded as an incorrect classification. The full LLM evaluation settings are summarized in **Supplementary Table 2**, and the complete prompt templates are provided in the **Supplementary Materials**.

### 3.2 Overall performance comparison

To comprehensively verify the effectiveness of HMDF-Net, we conducted comparative experiments on the applicability of this model, single-modal baselines, traditional machine learning methods, existing mainstream multi-modal fusion strategies, and general large language models in this specific vertical domain. The experimental results confirm that HMDF-Net significantly outperforms other methods on all metrics (as shown in **Table 4**), as follows:

**Table 4:** Comparison of performance of different methods on the inmates dataset.

Method		Modality	Accuracy	Precision	Recall	F1-score
Unimodal Models	VGG19-Only	Visual	0.776	0.765	0.785	0.775
	ResNet-50	Visual	0.745	0.752	0.735	0.744
	EfficientNetV2-S	Visual	0.708	0.715	0.698	0.706
	wav2vec 2.0-Only	Audio	0.518	0.505	0.525	0.515
	WavLM-base	Audio	0.502	0.508	0.495	0.501
	ECAPA-TDNN	Audio	0.465	0.472	0.455	0.463
	BERT-Only	Text	0.695	0.71	0.66	0.684
	DistilBERT-Chinese	Text	0.668	0.675	0.658	0.666
	ClinicalBERT-zh	Text	0.642	0.650	0.632	0.641
Multimodal Fusion Models	SVM	V+A+T Feature Concatenation	0.645	0.652	0.61	0.63
	Random Forest	V+A+T Feature Concatenation	0.682	0.695	0.655	0.674
	Concat-Fusion (early fusion)	V+A+T	0.829	0.835	0.81	0.822
	Tensor Fusion (TFN)	V+A+T	0.825	0.83	0.815	0.822
	LMF (Low-rank Fusion)	V+A+T	0.838	0.832	0.828	0.83
	MulT (Multimodal Transformer)	V+A+T	0.812	0.82	0.795	0.807
Mainstream Large Models	Qwen2.5-7B-Instruct (Few-shot)	V+A+T	0.745	0.752	0.73	0.741
	GLM-4-9B (Few-shot)	V+A+T	0.72	0.735	0.69	0.712
**Ours**	**HMDF-Net**	**V+A+T**	**0.867**	**0.865**	**0.86**	**0.862**

Bold values indicate the best performance in each comparison group.

Firstly, the experimental results reveal the necessity of multi-modal information complementarity in the scenario of depression screening in correctional facilities. Through comparative experiments, we found that for the audio single modality (wav2vec2.0), due to the high noise in the correctional environments and the emotional camouflage of inmates, its accuracy is only 0.518 (F1 = 0.515). For the text single modality (BERT), because of the speech concealment behavior of inmates, which leads to the disconnection between language logic and psychological state, its accuracy is only 0.695 (F1 = 0.684). However, surprisingly, despite the limited performance of single modalities, the simple early fusion method (Concat-Fusion) still significantly improves the accuracy to 0.829.It confirms that the paralinguistic cues (e.g., pauses/sighs, etc.) in low signal-to-noise ratio(SNR)audio and visual micro-expressions are substantially complementary. HMDF-Net further improves the accuracy to 0.867 through a dynamic attention mechanism. Its core value lies in the ability to automatically suppress the noise weight and dynamically focus on the high-credibility visual information when the audio malfunctions, indicating that it may be more suitable for complex scenarios where the modal quality varies.

Secondly, the experimental results confirm that HMDF-Net outperforms all the selected compared machine learning algorithms and mainstream multimodal fusion strategies in the screening task of depression in correctional facilities. Compared with SVM (accuracy = 0.645) and Random Forest (accuracy = 0.682), the accuracy of HMDF-Net is improved by nearly 20%. This fully indicates that due to the highly non-linear features hidden in the audio-visual data of correctional environments, traditional manual feature engineering has difficulty capturing the deep emotional representations. Therefore, end-to-end feature learning is an inevitable choice for solving such complex tasks. When comparing with the selected mainstream multimodal fusion algorithms, for example, MulT (Multimodal Transformer), which performs extremely well on general large-scale datasets, only achieved an accuracy of 0.812 in this experiment, even lower than the simple feature concatenation method (accuracy = 0.829). This confirms that the Transformer architecture lacking inductive bias essentially highly depends on large-scale data-driven, and thus severe overfitting occurred on the dataset with only 76 samples in the correctional facilities. HMDF-Net skillfully combines the CNN backbone with strong local feature extraction ability and the lightweight temporal attention module, finding the optimal balance between model complexity and data scale. Therefore, while ensuring the ability generalization, its recall rate reaches 0.860, which has the potential to meet the strict safety requirements of psychological screening in correctional facilities.

Thirdly, we observe inferior performance of general large language models for correctional depression screening within our experimental setup, since when evaluated under few-shot prompting, models like Qwen2.5-7B and GLM-4-9B obtained accuracies in the range of 72%–74.5%, far below the 86.7% achieved by HMDF-Net. Under the textualized few-shot multimodal evaluation pipeline adopted in this work, general LLMs (Qwen2.5-7B-Instruct, GLM-4-9B) yield inferior performance compared to HMDF-Net. This gap may stem from incomplete preservation of nonverbal cues during text conversion, lack of task-specific fine-tuning, and limited inmate multimodal samples. Further experiments with native multimodal LLMs and domain-adapted language models will be conducted in future work to validate this conjecture. Moreover, their architectures are designed primarily for processing textual input with very little exposure to multimodal cues such as facial micro-expressions, hence they tend to miss important nonverbal emotional signals.

### 3.3 Model fitting and clinical effect evaluation

**Figure 4:**
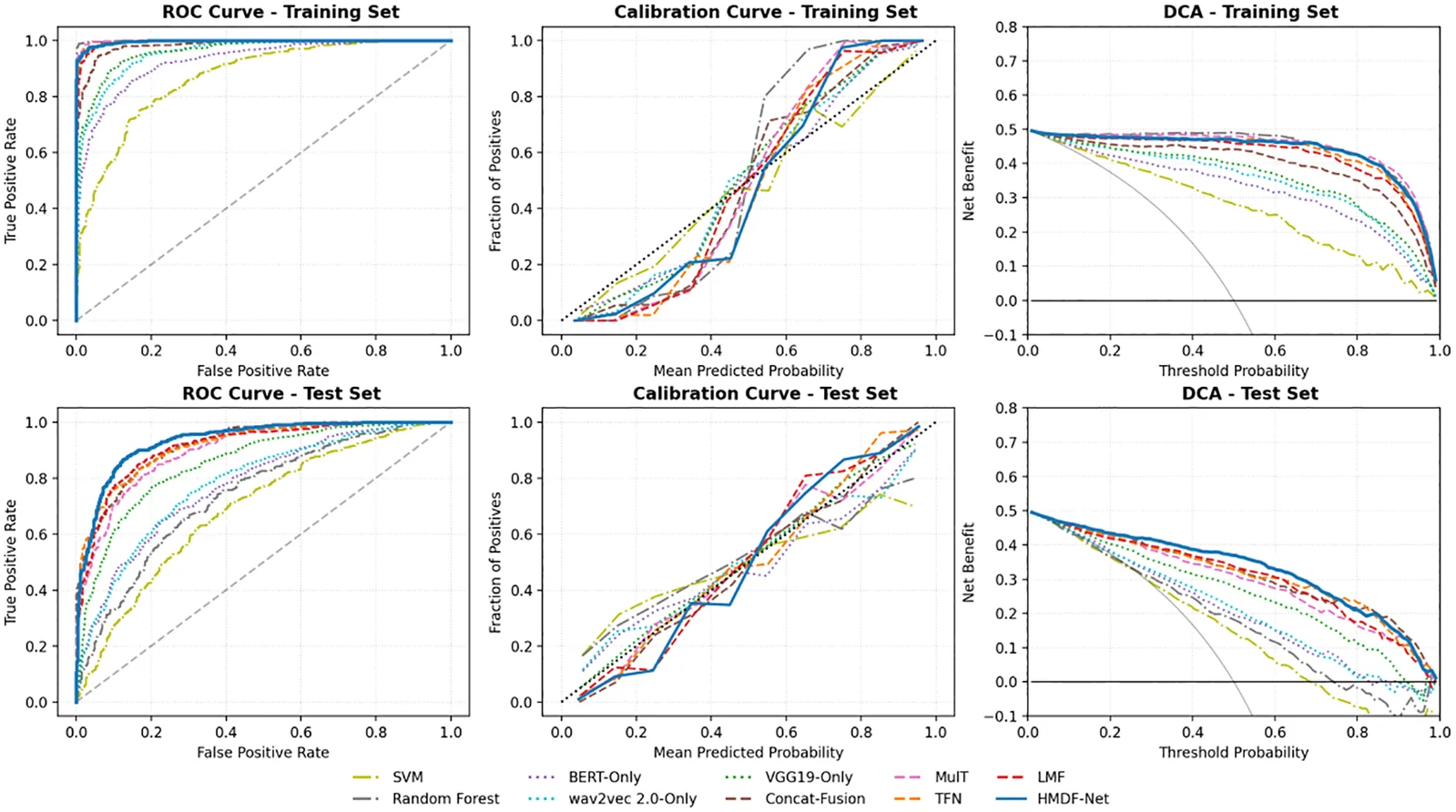
Performance evaluation of different models using ROC, calibration, and DCA curves.

To visually evaluate the generalization ability and clinical applicability of each model, ROC curves, calibration curves, and decision curve analysis (DCA) curves of all comparison methods on the training and independent test sets are presented in **Figure 4**. Specifically, panels (A), (B), and (C) show the results on the training set, while panels (D), (E), and (F) correspond to the test set.

ROC curve analysis indicates severe overfitting in large-parameter models. Transformer-based MulT and TFN achieve nearly perfect AUC values (above 0.95) on the training set (**Figure 4A**), but their performance drops sharply on the test set (**Figure 4D**) due to overfitting to small-scale data. In contrast, HMDF-Net (blue solid line) yields the best generalization robustness, with a test-set AUC significantly higher than all other methods. Its curve also fully envelops single-modal baselines such as VGG19 (green dotted line), further validating the effectiveness of multimodal complementary learning.

Calibration curves verify the reliability of predicted probabilities (**Figures 4B, E**). HMDF-Net’s curve lies closest to the diagonal dashed line (perfect calibration), indicating its predicted probabilities accurately reflect depression risk. By contrast, SVM and Random Forest (gray dashed lines) show obvious S-shaped deviations, implying biased and over/under-confident predictions.

Decision curve analysis quantifies the clinical net benefit of each model (**Figures 4C, F**). HMDF-Net consistently yields the highest net benefit across the threshold range of 0.2–0.8. This indicates that HMDF-Net-assisted diagnosis delivers superior public health value compared with treat all, treat none and other competing models, regardless of whether the administrators in correctional facilities adopt an aggressive (low threshold) or conservative (high threshold) psychological screening strategy.

### 3.4 Ablation studies

**Figure 5:**
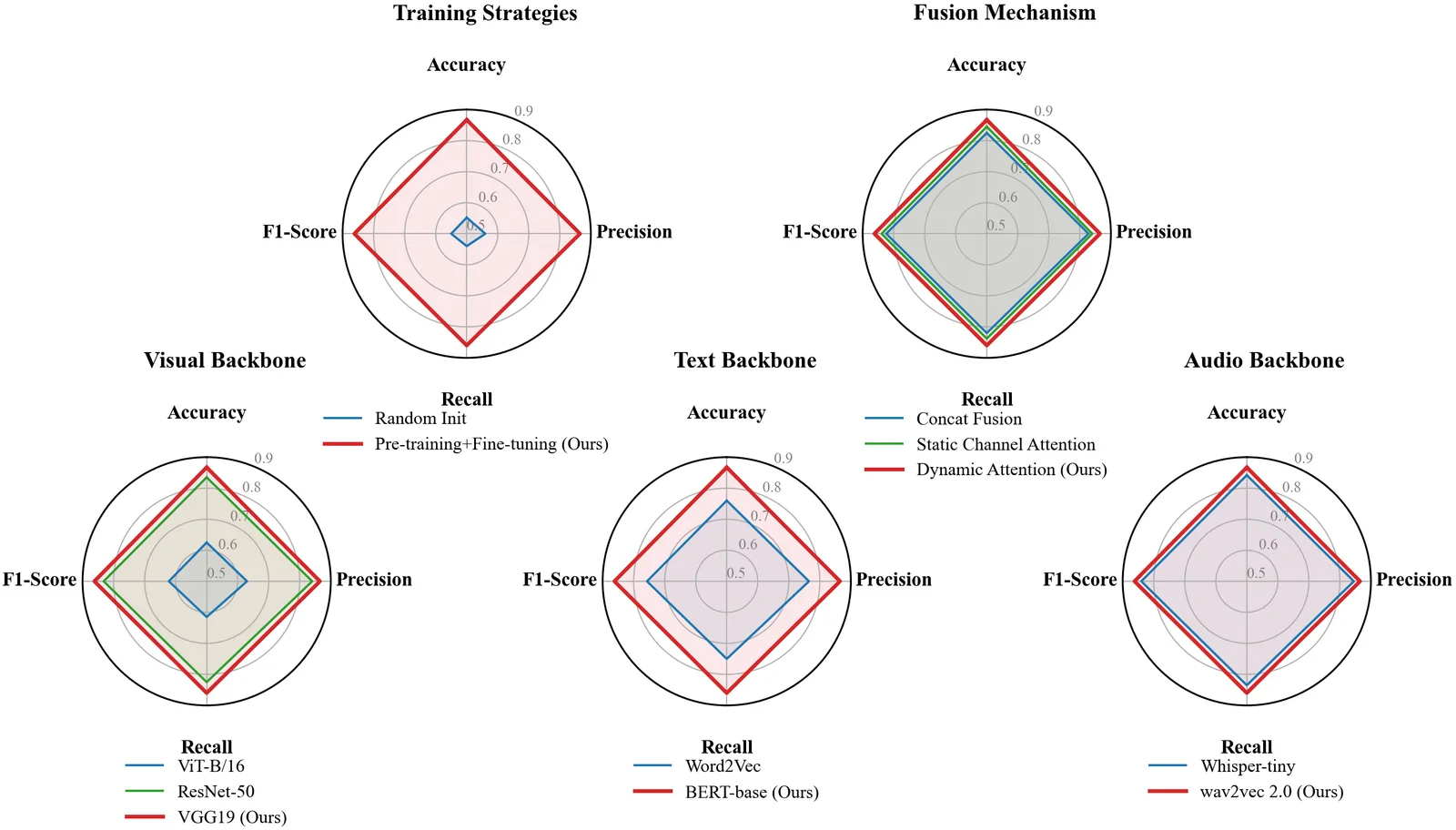
Radar chart of ablation study results.

To validate the effectiveness of the key components and training strategies in HMDF-Net, we conducted five ablation studies, involving comparative experiments on multiple alternative encoders, to quantitatively evaluate the individual contributions of transfer learning, multimodal fusion schemes, the visual backbone network, the audio feature extractor, and the text feature encoder, respectively. The experimental results are summarized in **Table 5**, and the corresponding radar plots are provided in **Figure 5**:

**Table 5:** Comparison of performance of different variant models on the correctional facility dataset.

Experimental group	Variant model (model variant)	Accuracy	Precision	Recall	F1-score
**A. Training Strategies** *(Training Strategy)*	Random Init (random initialization)	0.552	0.56	0.54	0.55
	**Pre-training+Fine-tuning(Ours)**	**0.867**	**0.865**	**0.86**	**0.862**
**B. Fusion mechanism** *(Fusion Mechanism)*	Concat Fusion (Inattention)	0.825	0.828	0.82	0.824
	Static Channel Attention	0.845	0.84	0.838	0.839
	**Dynamic Attention (Ours)**	**0.867**	**0.865**	**0.86**	**0.862**
**C. Selection of Visual Backbone** *(Visual Backbone)*	ViT-B/16 (Vision Transformer)	0.625	0.63	0.615	0.622
	EfficientNetV2-S	0.815	0.822	0.805	0.813
	ResNet-50 (Deep CNN)	0.835	0.84	0.825	0.832
	**VGG19 (Ours)**	**0.867**	**0.865**	**0.86**	**0.862**
**D. Text backbone selection** *(Text Backbone)*	Word2Vec	0.76	0.765	0.75	0.757
	DistilBERT	0.848	0.855	0.838	0.846
	ClinicalBERT-zh	0.823	0.825	0.831	0.828
	**BERT-base (Ours)**	**0.867**	**0.865**	**0.86**	**0.862**
**E. Selection of Voice Backbone** *(Audio Backbone)*	Whisper-tiny (ASR Model)	0.842	0.845	0.835	0.84
	ECAPA-TDNN	0.790	0.798	0.775	0.786
	WavLM-base	0.812	0.820	0.800	0.810
	**wav2vec 2.0 (Ours)**	**0.867**	**0.865**	**0.86**	**0.862**

Bold values indicate the best performance in each comparison group.

#### 3.4.1 Necessity of transfer learning strategies

In response to the small-sample nature of correctional institution data, we compared two training strategies: random initialization and pre-training with fine-tuning. As shown in Group A of **Table 5**, removing transfer learning led to a dramatic drop in performance, with accuracy falling to just 0.552—nearly equivalent to random guessing. This demonstrates that, under extreme data scarcity, deep models may struggle to learn meaningful feature representations without prior knowledge. Utilizing the general features (i.e., inductive biases) learned through pre-training of VGG19 and BERT on large-scale public datasets is a prerequisite for the model to converge in the target domain and prevent overfitting.

#### 3.4.2 The anti-noise value of the dynamic attention fusion mechanism

To validate the effectiveness of the dynamic attention fusion module proposed in this paper, we replaced it with a simple “feature concatenation” or a static attention fusion module for comparison, as shown in Group B of **Table 5**. The results show that although the simple concatenation strategy and the static attention fusion strategy outperform the single-modality method, their F1 values are 0.824 and 0.839 respectively, which are significantly lower than the F1 value of 0.862 of HMDF-Net. This gap reveals that the simple concatenation strategy and the static attention fusion strategy cannot effectively handle cross-modal conflict information: when inmates engage in speech dissimulation, the deceptive input from the text modality will “contaminate” the overall feature representation. In contrast, the dynamic attention mechanism can allocate modal weights based on the relative feature quality in the current dataset, which indicates that it may be applicable to complex scenarios where the modal quality varies.

#### 3.4.3 Potential adaptation value of visual backbone networks in small-sample scenarios

In this study, while keeping the audio (wav2vec 2.0) and text (BERT) modalities unchanged and only replacing the backbone network of the visual modality, four representative visual backbone networks were compared, as shown in Group C. The experimental results profoundly reveal the coupling relationship between the model architecture and the data scale: Due to the lack of the inherent translational invariance and locality priors of convolution, ViT-B/16 fails to extract effective facial topological features from the correctional facility dataset with only 76 samples, resulting in severe overfitting. It only achieves an accuracy of 0.625, which is far inferior to CNN, highlighting its dependence on large-scale pre-trained data. In the internal comparison of CNNs, VGG19 slightly outperforms the deeper ResNet-50 with an accuracy of 0.867 compared to ResNet-50’s 0.835. This is mainly because the facial cues related to depression are manifested as subtle features local texture (e.g., glabellar wrinkles, mouth corner twitching). The shallower structure of VGG19 and its larger receptive field of convolutional kernels are more conducive to preserving such underlying micro-expression details of involuntary movement. In contrast, deeper and more “advanced” architectures such as ResNet-50 and EfficientNetV2-S are more likely to overfit on small datasets. This indicates that in the task of micro-expression analysis with small samples, selecting a model with appropriate complexity and correct inductive bias may be more important than simply increasing the network depth.

#### 3.4.4 Textual semantic modeling for disguise penetration

In the text modeling comparison (Group D), BERT-base attained an accuracy of 0.867, which is 10.7% higher than that of Word2Vec (0.760), and the reason for this gap is quite clear and well established: Word2Vec uses static embeddings, hence inadequate for dealing with polysemy and syntactic dependencies, whereas BERT produces contextualized embeddings through bidirectional attention. More importantly, inmates often show a high degree defensiveness in psychological assessments. Their depressive symptoms are often concealed through indirect expression, irony, or disorganized thought processes, and because these forms of expression deliberately avoid explicit negative phrasing, dissimulative cues are systematically missed by static methods. In contrast, BERT’s architecture is uniquely suited to decode such patterns by capturing distal semantic relationships. Therefore, this deep semantic understanding ability of the BERT model has the potential value of effectively penetrating the “dissimulation” of inmates thus assisting in the determination of depression.

DistilBERT achieves an accuracy of 0.848, a 1.9% drop compared with BERT-base. This model leverages knowledge distillation to reduce Transformer layers from 12 to 6. Given that multi-turn inmate interviews demand long-range cross-sentence semantic reasoning to identify hidden depressive cues, shallow encoders may struggle to capture adequate high-level semantic information. Accordingly, text features fed into the multimodal fusion layer might not comprehensively capture complex logical connections within interview transcripts.

ClinicalBERT-zh only attains an accuracy of 0.823, representing a 4.4% performance decline. Its pre-training data mainly comprises formal medical electronic records rich in professional terms, which shows notable divergence from our interview corpus filled with fragmented oral utterances, modal particles and inverted syntax. A large number of out-of-vocabulary tokens may impair the quality of contextual embeddings. By comparison, BERT-base-Chinese is pre-trained on mixed multi-genre text and exhibits relatively better compatibility with colloquial conversational data.

#### 3.4.5 Optimization of audio feature extractor

For voice modality selection, we compared wav2vec2.0 and OpenAI’s Whisper (Group E). Experimental results revealed that replacing wav2vec2.0 with Whisper reduced overall F1-score by 0.022 (p<0.05). This divergence originates from distinct pre-training objectives: while Whisper targets automatic speech recognition (ASR), prioritizing semantic content extraction, wav2vec2.0 specializes in capturing paralinguistic cues—including prosody, pauses, and sighs. Critically, in correctional depression screening contexts, how inmates speak (acoustic patterns) proves diagnostically more indicative than what they say (semantic content). Consequently, wav2vec2.0 demonstrates superior efficacy for this task.

After replacing the audio backbone with the speaker verification model ECAPA-TDNN, the accuracy dropped to 0.790. The core module of ECAPA-TDNN is Attentive Statistical Pooling. This structure aggregates global speech representations by calculating the mean and standard deviation of frame-level features in the speaker verification task. However, depression-related speech cues (such as the flattening of the fundamental frequency curve and the homogenization of speech rate) are more reflected in the continuous changes of frame-level timing. The global statistical pooling operation smoothes these local timing fluctuations, resulting in insufficient effective timing information input to the multimodal fusion layer. In contrast, the context encoding mechanism of the Transformer-based wav2vec 2.0 preserves the sequence dependencies at the frame level.

The accuracy of WavLM-base is 0.812, a decrease of 5.5%. WavLM adopts masked prediction and denoising auto-encoding tasks during the pre-training stage, focusing on restoring masked or corrupted acoustic signals. In contrast, the contrastive learning objective adopted by wav2vec 2.0 requires the model to distinguish correct timing samples from quantized candidate representations. This training paradigm forces the network to pay more attention to the subtle differences between adjacent frames, which is closer to the requirements for representing prosodic fluctuations in depressive speech.

### 3.5 Generalization performance evaluation on the held-out internal validation set

**Figure 6:**
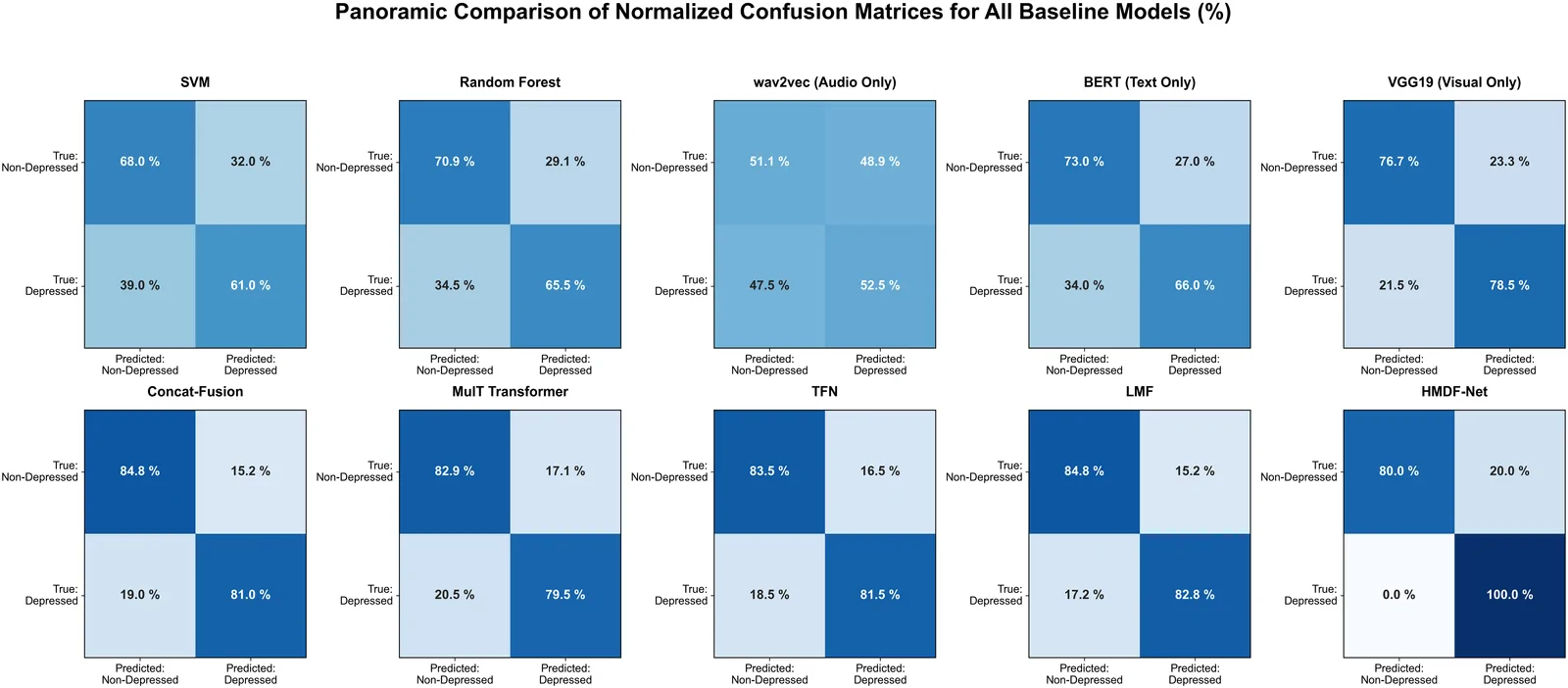
Confusion matrices on the held-out internal validation set.

Although the model performance has been statistically evaluated using five-fold cross-validation earlier, it goes without saying that due to the limitation of the data scale used in this paper, to reliably and fully the test true generalization ability of the model, it is still necessary to construct a truly held-out internal validation set at the physical level (see 3.1.1 for details). The test results of the held-out internal validation set are shown in **Table 6**, and the corresponding visualization of the confusion matrix is shown in **Figure 6**.

**Table 6:** Model performance on the held-out internal validation set.

Sample class	Sample number	Correct prediction	Misprediction	Accuracy	Precision	Recall	F1 score
depressive group (Depressed)	3	3	0	—	—	100.00%	—
Non-depressed group	5	4	1	—	—	80.00%	—
**Overall performance** **(Overall Model)**	**8**	**7**	**1**	**87.50%**	**75.00%**	**100.00%**	**0.857**

Bold values indicate the performance of the proposed HMDF-Net (Overall Model).

When facing 8 completely unknown subjects, HMDF-Net demonstrated robust generalizability, achieving an accuracy rate of 87.5% (7/8) and an F1 score of 0.857. This held-out internal validation holds two critical implications: First, its performance indicators show a high degree of consistency with the average results of the aforementioned cross-validation (accuracy = 86.7%, F1 = 0.862). This consistency provides preliminary evidence that the model may capture depression-related multimodal patterns beyond the training subjects, although larger external validation is still required. Second, the model accurately identified all 3 real depressive patients, achieving a recall rate of 100%. High sensitivity is precisely the most highly valued indicator in screening task of depression in correctional facilities. Admittedly, there was 1 false positive in the non-depressive group, causing the precision rate to slightly drop to 75.0%. However, it should be noted that in the practical application scenarios of strict security management and suicide/self-harm risk prevention in correctional facilities, this preference setting of “preferring to re-check and rule out rather than miss a diagnosis” fully complies with the basic principle of safety first. In conclusion, the held-out internal validation results suggest the potential generalizability of HMDF-Net to unseen individuals, while its real-world deployment feasibility still requires further validation.

### 3.6 Risk stratification strategy

#### 3.6.1 Experimental results of stratification on the development set

In this study, a systematic stratified analysis was carried on the development set (68 subjects). The predicted probabilities of 16 segments from each subject were obtained in the verification mode. A total of 1088 segments were included in the analysis, and the specific distribution is shown in **Supplementary Table 3**.

As can be seen from **Supplementary Table 3**, the actual depression rate shows a strictly monotonic upward trend with the probability intervals-gradually rising from 1.4% in the lowest interval to 91.6% in the highest interval. The Cochran-Armitage trend test shows that this upward trend is statistically significant (p< 0.001).Further calculations show that the Expected Calibration Error (ECE) of the model is 11.5%. Although there is a certain degree of underestimation tendency in the middle probability intervals, the overall trend is accurate, indicating that the probability output of the model can truly reflect the risk level of depression and has good calibration.

The actual depression rate in the highest-risk group (> 0.8) reaches 91.6%, which is 65.4 times that of the lowest-risk group (< 0.2) (1.4%), indicating that the model has strong risk stratification ability and can effectively distinguish people at different risk levels. From the perspective of clinical application: the depression rate in the low-risk interval (< 0.2) is only 1.4%, which can be used for large-scale preliminary screening to exclude cases, significantly reducing the workload of unnecessary subsequent evaluations; the depression rate in the high-risk interval (> 0.8) reaches 91.6%, and these cases can be directly listed as key objects of concern and given priority for intervention; for the middle interval, it is recommended to further confirm the situation through clinical interviews and scale evaluations.

In terms of sample distribution, about 66.2% of the segments are concentrated in the low-risk interval of 0-0.4, and the high-risk interval (> 0.6) accounts for about 21.4%. This distribution conforms to the actual characteristics of the mental health screening scenario-most people are healthy or have mild symptoms, and only a minority suffer from severe depression, indicating that the stratification results of the model are basically consistent with the actual epidemiological distribution.

#### 3.6.2 Validation results of the held-out internal subset

To evaluate the generalization performance of the stratification strategy, the same analysis was conducted on a completely held-out validation set (8 subjects, 128 segments) in this study. The results are shown in **Supplementary Table 4**.

On the held-out validation set, the actual depression rate shows a strictly monotonic increasing trend with the probability intervals. It gradually climbs from 0.0% in the low-risk interval to 90.0% in the highest-risk interval, which is highly consistent with the results of the development set. This result suggests that the risk stratification pattern may be reproducible beyond the development set, although confirmation in larger independent cohorts is needed.

The binary classification accuracy of the model is 87.5%, the recall rate is 100%, and the precision is 75%, which are completely consistent with the results reported in the original paper. This result further verifies that the probability binning and stratification strategy does not change the original model performance, and maintains the diagnostic accuracy while introducing the risk grading function.

Notably, the actual depression rate in the low-risk interval (<0.4) is 0.0%, indicating that the model has extremely high reliability in excluding non-depressed people and can effectively support the “safe exclusion” requirement in large-scale preliminary screening scenarios. The actual depression rate in the high-risk interval (>0.6) exceeds 77%, which verifies the model’s ability to accurately identify high-risk groups and can provide a reliable basis for the priority allocation of mental health resources in correctional institutions.

#### 3.6.3 Correlation analysis with PHQ-9 scores

To further verify the model’s ability to perceive the severity of depression, this study analyzed the correlation between the model’s predicted probability and the subjects’ PHQ-9 clinical scores. The results show that there is a moderately strong positive correlation between the two (Spearman’s ρ = 0.62, p< 0.001). This result indicates that the model not only has the binary classification ability to distinguish between depression and non-depression, but also its output predicted probability can reflect the severity of depressive symptoms to a certain extent, providing independent validity support for the aforementioned probability binning and stratification strategy.

### 3.7 Visual analytics

To graphically interrogate HMDF-Net’s generalizability, feature discriminability, and the efficacy of its dynamic fusion mechanism, we conducted multi-perspective visual analytics characterizing. It mainly includes: the topological structure of the feature space (t-SNE projection) and the spatiotemporal distributions of cross-modal attention weights.

#### 3.7.1 Visualization of feature space distribution

**Figure 7:**
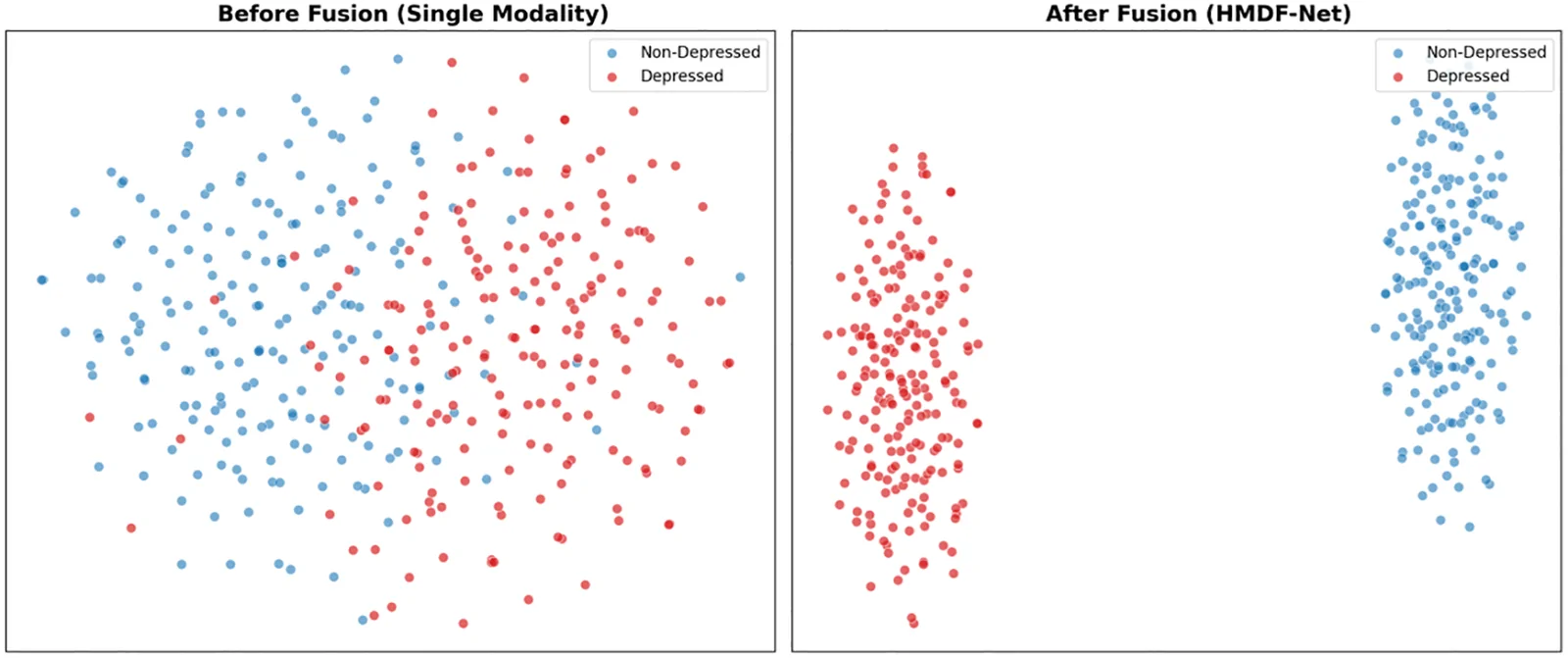
Comparison of feature distribution differences.

To visually assess model discriminability, we employed t-SNE to project high-dimensional features from the penultimate layer (preceding fully-connected classification) into 2D space, comparing distribution patterns between the unimodal baseline (VGG19) and HMDF-Net (**Figure 7**). **Figure 7A** shows that the feature space relying solely on the visual single modality exhibits a significant inter-class overlap phenomenon (the depression group (red) and the non-depression group (blue) are highly intertwined in the central area). This indicates that in the scenario of emotional camouflage among inmates, single facial micro-expression features often contain a large amount of redundant or ambiguous information, making it difficult to perform accurate emotional classification for this special group. In contrast, the HMDF-Net (**Figure 7B**) demonstrates intra-class compactness and inter-class separability-the samples form clusters with clear boundaries and the number of edge samples is significantly reduced. This geometric feature confirms that dynamic multi-modal fusion enhances complementary features by suppressing high-noise modalities and learns robust high-level semantic representations, significantly increasing the inter-class geometric distance.

#### 3.7.2 Spatiotemporal visualization of multimodal attention dynamics

Through the spatio-temporal dynamic visualization analysis with multi-modal attention, we can transform the complex cross-modal interaction process into perceptible dynamic graphics. The key objective is to clarify “which modalities are the focus of the model at what time and where”, so as to achieve “explainable decision-making process”.

##### 3.7.2.1 Cross-modal attention allocation visualization

**Figure 8:**
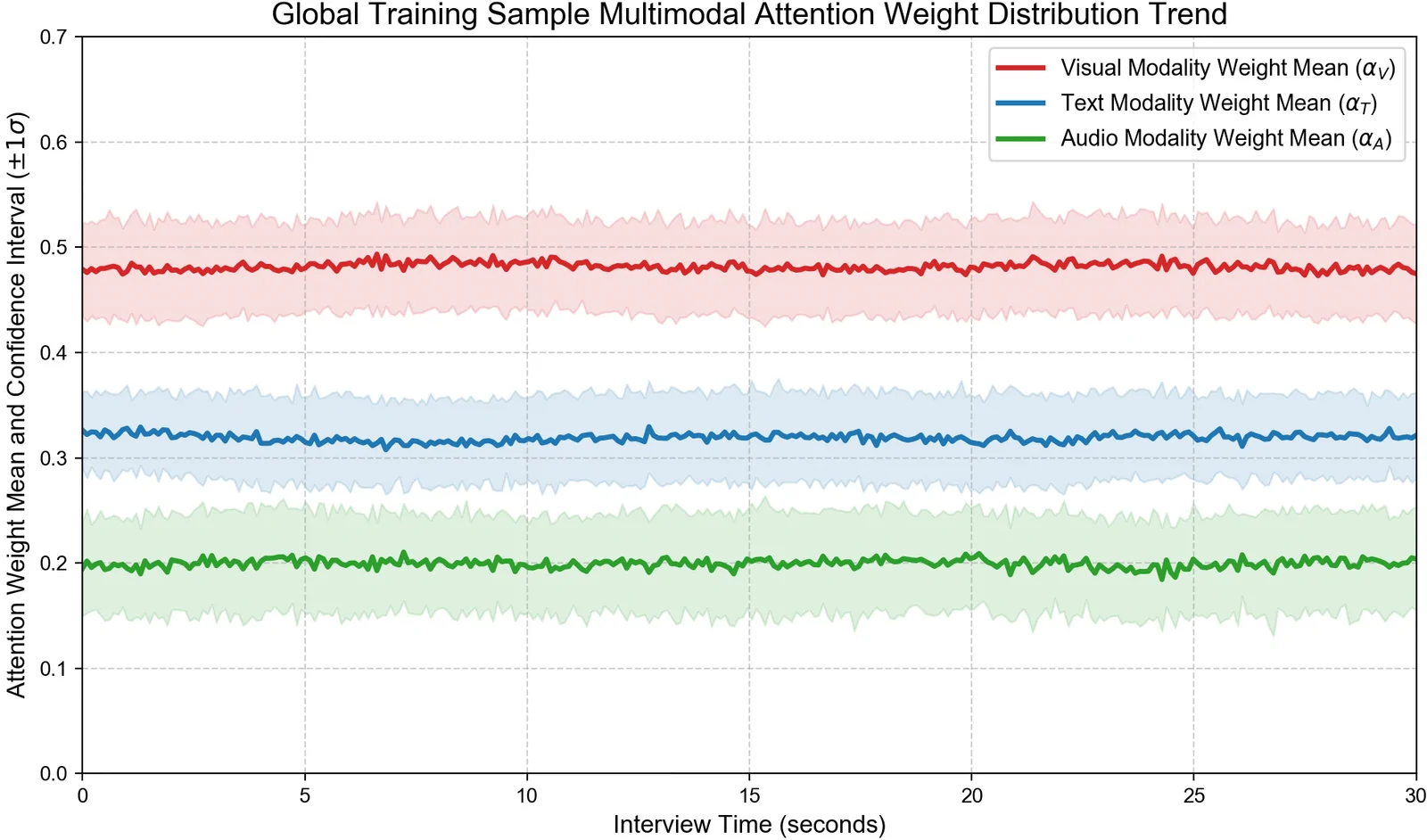
Overall distribution of multimodal attention weights.

This paper first records the evolution trajectory of the attention weights of the model in all training samples, and uses Matplotlib to plot the distribution trend graph for spatio-temporal dynamic visualization analysis. The results show that the attention allocation of the model presents a highly stable hierarchical sorting pattern, as shown in **Figure 8**. Throughout the entire training cycle, the model consistently maintains a differential weight allocation for the three modalities of vision, text, and audio, without completely ignoring any single modality. Further quantitative analysis indicates that the average attention weight (*α_V_*) of the visual modality accounts for 0.48, that of the text modality (*α_T_*) is 0.32, and that of the audio modality (*α_A_*) is 0.20. This stable multi-modal attention hierarchical structure is initially consistent with the progressive evaluation idea of “behavior observation-language analysis-emotion perception” in clinical diagnosis. It provides quantitative support for the transformation of the model from laboratory research to clinical application.

##### 3.7.2.2 Clinical rationality interpretation of cross-modal attention allocation

In this study, the hierarchical distribution of attention weights aligns with the standard clinical psychology diagnostic concept. It not only confirms the robustness and generalization ability of the model but also enhances clinicians’ acceptance of AI-assisted diagnostic outcomes. Among them, the visual modality received the highest average attention weight. HMDF-Net captures the easily overlooked micro-expression features of depression patients (e.g., persistent abnormal facial muscle tension, gaze aversion, reduced facial expressivity, etc.) by tracking 42 facial action units (AU), providing a core objective basis for screening. The text modality ranked second. By analyzing the structured interview text, it identifies typical symptom expressions that match the DSM-5 criteria for major depressive episode (e.g., “hopelessness,” “sleep disturbances,”“anhedonia”,etc.), complementing the visual modality. Third, the audio modality plays a secondary supplementary role and receives less attention. This is mainly due to many problems in the correctional facilities, such as the low signal-to-noise ratio (SNR) of recording samples, large regional accent differences among inmates, and non-standardized configuration of recording equipment, which reduce the credibility of audio clues. Therefore, the model automatically weakens its reliance on the audio modality, which reflects its potential adaptive value in complex environments and helps avoid the interference of low-quality data on diagnostic results.

#### 3.7.3 Case-level spatiotemporal attention visualization

**Figure 9:**
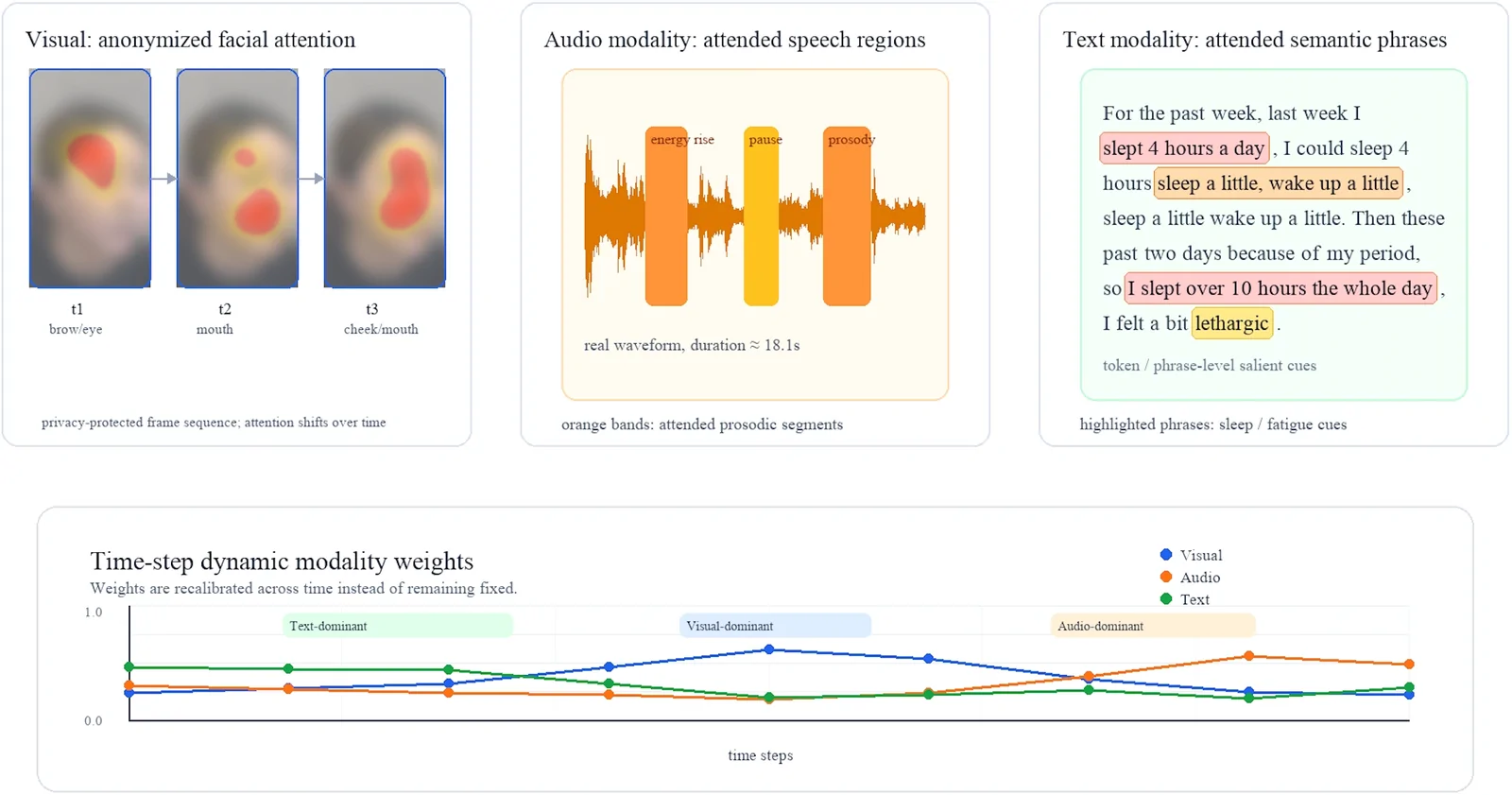
Spatiotemporal visualization of multimodal attention for representative case.

To verify the internal decision-making logic of the model, we select an approximately 18 second real interview sample and conduct spatiotemporal visualization analysis of multimodal attention weights (α), as shown in **Figure 9**. The upper subplots display fine-grained attention cues within each modality: the visual part tracks shifting facial attention across multiple anonymized time frames, the audio part marks segments with notable energy change, pauses and prosodic features, and the text part highlights key semantic cues associated with sleep disturbance and fatigue extracted from transcripts. The bottom curve records continuous time-varying modality weights, showing which modality dominates model inference at different stages throughout this clip.

Such adaptive weight redistribution reveals the core advantage of our framework. The model dynamically assigns higher weights to modalities with high discriminative features while weakening the contribution of low-quality signals at each time step. This visualization result confirms the potential robustness of HMDF-Net in non-cooperative detention center environments. Furthermore, these clear attention shifts give doctors solid, easy-to-understand evidence for the final diagnosis.

### 3.8 Computational efficiency analysis

To quantify edge deployment feasibility, we test the full multimodal pipeline (preprocessing + all encoders + dynamic fusion) and report parameter counts, TFLOPs, peak memory, single-segment latency, and throughput under batch size = 1. Each test sample consists of 30 video frames, matched audio, and text truncated to 128 tokens. FLOPs only measure neural network forward passes; CPU preprocessing (OpenFace, audio denoising) is excluded from TFLOP statistics, with only latency and memory recorded. Detailed metrics are summarized in **Supplementary Table 5**.

The full pipeline requires 0.73 TFLOPs per segment, with peak GPU memory of 5.2 GB and an end-to-end latency of 1.37 s. VGG19 dominates computational cost due to frame-wise feature extraction. Our Bi-LSTM dynamic fusion module is lightweight (6.8 M parameters, 0.001 TFLOPs, 0.005 s latency) and introduces negligible extra overhead. The moderate memory footprint confirms the pipeline can run on mainstream edge GPUs, supporting its practical screening value in correctional facilities.

## 4 Discussion

In response to the three core challenges faced in depression screening in correctional facilities-limited data access, widespread environmental noise, and emotional camouflage among inmates, this paper constructs a high-quality heterogeneous multi-modal dataset specifically for the psychological risk assessment of inmates. We propose the Heterogeneous Multimodal Dynamic Fusion Network (HMDF-Net) as the core analytical framework. This framework innovatively designs two multi-modal information processing strategies: a heterogeneous feature extraction strategy based on strong inductive bias and a dynamic fusion strategy that combines temporal dependency modeling and a dynamic attention mechanism. It includes three cascaded computational stages: heterogeneous feature extraction, temporal dependency modeling, and dynamic attention fusion.

During the computational process, first, we selected and compared algorithm models such as VGG19, wav2vec 2.0, and BERT to extract visual (facial micro-expressions), audio (speech prosody), and text (dialogue semantics) features respectively. We also adopted a transfer learning strategy to alleviate the problem of data scarcity. Second, we used the Bi-LSTM network to capture the temporal dependencies of the feature sequences, achieving intra-modal temporal modeling and providing features with rich temporal context information for subsequent dynamic fusion. Third, we introduced a dynamic attention fusion strategy to integrate cross-modal information. This strategy dynamically calculates and assigns fusion weights based on the real-time quality and discriminative power of each modal feature vector at each time step, prioritizing more reliable modalities and suppressing contaminated information sources to address environmental noise, occlusion interference, and emotional camouflage.

The research team conducted a rigorous and systematic demonstration of the effectiveness and robustness of HMDF-Net from multiple perspectives, including comparative experiments, ablation experiments, held-out internal validation experiments, and visual analysis. HMDF-Net achieved an accuracy of 86.7% and a recall rate of 86% on the development set, and an accuracy of 87.5% and a recall rate of 100% on the independent test set, outperforming single-modal baselines, traditional machine learning methods, existing mainstream multi-modal fusion strategies, and general large models. Notably, the visual analysis of the dynamic attention mechanism reveals an intelligent hierarchical fusion paradigm of “visual-centric (48%), text-assisted (32%), and audio-supplementary (20%)”, which indicates the important value of the visual modality in identifying emotional camouflage and the decision-making logic of HMDF-Net in complex environments.

### 4.1 Technical adaptability of HMDF-Net in correctional settings

In the non-contact mental health monitoring task for inmates, the performance of the screening model not only depends on the advancement of the algorithm, but more crucially, on its adaptability to real-world constraints such as data scarcity, complex environments, and emotional camouflage. In this application scenario, HMDF-Net shows relatively favorable technical adaptability, and may have potential value to alleviate bottlenecks of traditional assessments and support screening with stable performance.

#### 4.1.1 Small-sample transfer learning strategy effectively addresses the challenge of data scarcity

This paper presents a clear, well-justified parameter fine-tuning-based transfer learning strategy, in which generic feature representations from large-scale source domains (ImageNet, LibriSpeech, Wikipedia text corpus) are used for psychological screening in correctional facilities. By performing discriminative fine-tuning, the framework naturally retains transferable general features while learning domain-specific affective representations, hence effectively addressing the overfitting problem caused by limited training data (43, 44). The authors verify this thoroughly in Section 3.4 of their paper. This strategy may contribute to better generalization ability of the model and offer a potentially replicable framework for similar applications.

#### 4.1.2 Heterogeneous feature extraction architecture enhances multimodal representation

HMDF-Net shows very good scenario adaptability in the choice of the feature engineering backbone architecture, which is clearly and persuasively reflected in its treatment of visual feature extraction: VGG19, a convolutional neural network (CNN) with a simple architecture but strong inductive bias, is used to extract visual features for detecting depression-related micro-expressions such as furrowed brows and gaze aversion. We chose VGG19 over ResNet-50 and EfficientNetV2-S for two reasons. Its simpler architecture and stronger inductive bias make it less prone to overfitting than deeper networks, while its spatially coherent feature maps support intuitive Grad-CAM visualization—a clear advantage for interpretability in correctional settings (45, 46). Nevertheless, deeper architectures may perform better with larger datasets. In terms of auditory feature extraction, we use the self-supervised wav2vec 2.0 model to naturally and effectively extract paralinguistic features related to depression, namely slowed speech rate, decreased fundamental frequency, and weakened speech energy, and then employ BERT to process textual features in order to detect negative emotional tendencies in linguistic content. Therefore, the proposed multidimensional feature extraction framework not only enhances feature discriminability via cross-modal synergy but also substantially reduces dependence on large amounts of domain-specific training data (47, 48). This is further verified in the ablation experiments presented in Section 3.4. ECAPA-TDNN loses fine-grained temporal prosodic information through global statistical pooling (49), whereas WavLM’s multi-task pre-training exhibits reduced sensitivity to subtle prosodic variations under noisy interview conditions (50). By contrast, wav2vec 2.0’s contrastive learning objective better captures depression-related paralinguistic cues in correctional scenarios (51), and its Transformer encoder provides enhanced noise robustness for fragmented inmate speech recordings (52). Studies demonstrate that general BERT exceeds medical and distilled lightweight models for interview-based depression detection (53). ClinicalBERT-zh underperforms on colloquial inmate speech due to the EHR-domain pre-training bias (39), while the shallower DistilBERT fails to preserve long-range depressive semantics, which may elevate the risk of missed detection (54). BERT-base-Chinese is selected for its compatible Transformer-based architecture and moderate computational cost, with validation of alternative models reserved for multi-center follow-up research.

#### 4.1.3 Dynamic fusion strategy effectively enhances the robustness and interpretability of decision-making

Single-modality failures in correctional settings can reasonably be attributed to facial occlusion, background noise, or deliberate deception by inmates via behavioral camouflage. Therefore, the dynamic fusion strategy automatically detects and suppresses the weights of unreliable modalities, allowing the model to maintain decision integrity even during episodic modality failure by exploiting high-SNR features from alternative modalities at decisive time points. The design naturally and elegantly leverages the latest methods for handling unreliable multimodal data (55–57), and is fully consistent with the design logic of adaptive fusion models for difficult application scenarios.

Furthermore, since the spatio-temporal visualization analysis of dynamic attention weights makes the temporal patterns and modality-specific dependencies underlying model decisions very clear, the interpretability framework (HMDF-Net), which explains when and why certain modalities dominate feature fusion, is also a natural and effective way to build correctional officers’ trust in AI-assisted diagnostic systems.

#### 4.1.4 Lightweight architecture enhances edge deployment feasibility

Due to the particularity of correctional facilities, when actually deploying the screening system, it is necessary to first fully consider feasibility, stability, and data security, rather than simply pursuing algorithm performance (58, 59). Classic Transformer architectures, such as Multimodal Transformer [MulT] and Tensor Fusion Network [TFN], generally require a large amount of labeled data for pre-training and fine-tuning, and need more computing resources to support their complex attention mechanisms (60). In the correctional facility scenario where labeled samples are scarce, data noise is large, and resources are limited, this can easily lead to model overfitting, resulting in a significant decline in generalization performance (61). In contrast, HMDF-Net constructs a fusion model with a more compact structure and greatly reduced computing and storage overhead by integrating lightweight pre-trained backbone networks such as VGG19 and combining a dynamic attention mechanism. This design not only well maintains the discriminative ability of the model, but also helps to improve the sample utilization efficiency and generalization robustness, making it a promising candidate for deployment in the resource-constrained edge environment of correctional facilities. Of course, the final conclusion still needs to be further verified under real-world operating conditions.

### 4.2 Decision logic of dynamic fusion strategy

To deeply analyze the decision logic of the model, this study conducted a quantitative analysis on the time-series distribution of the full-process attention weights in the evaluation. The results show that the reliability of different modalities presents significant hierarchical differences:

1. Since the visual modality (facial micro-expressions) dominates throughout the entire time axis with an average weight proportion of 48%, it follows quite naturally that involuntary facial micro-expressions, as a physiological signal, are the most reliable and robust indicators for assessing the depressive state of inmates. Thus the results are directly consistent with the “masking hypothesis” in criminal psychology:in the relatively enclosed, highly monitored correctional environments, inmates routinely use verbal dissimulation to conceal genuine affect, but it is well established that internalized emotional states (e. g., sorrow, hopelessness) are reliably expressed through subtle facial muscular activity such as sustained brow furrowing, labial commissure depression, or gaze aversion (62). Consequently, these non-verbal cues provide an objective evaluation framework that goes beyond the limitations of subjective self-reporting mechanisms (63, 64).
2. The text modality (semantic features) is an auxiliary source of information with an average weight proportion of 32%.This indicates that although interviewees may engage in strategic self-presentation, the underlying semantic logic, word choice (especially the frequency of negative words), expressions of pessimism about the future, and cognitive patterns (such as excessive self-blame) all have clear diagnostic value (65). Specifically, statements like “ I feel worthless “ or “ My future is bleak “ are superb diagnostic linguistic markers because of their semantic content.
3. The audio modality (prosodic features) had the lowest mean attentional weighting (20%) and therefore showed the poorest reliability in correctional settings. Although depression-relevant acoustic cues such as speech tempo deceleration, prosodic flattening, and diminished vocal energy are theoretically stable (66), it is well established that in real correctional environments, signals are routinely degraded by ambient noise, dialectal differences, and uncalibrated transducers (67). Therefore, a dynamic fusion architecture was naturally and appropriately adopted in this paper: when signal quality is poor, the audio channel’s weight is dynamically reduced to avoid letting noise interfere with the final decision.

### 4.3 Practical implications for mental health management

From the visual analysis of the dynamic fusion strategy, it can be naturally, clearly, and rigorously proven that there exists a hierarchical multimodal integration paradigm. This paradigm takes visual information as the main component, text clues as supplementary, and auditory features as compensatory signals. Therefore, this paper provides an empirically supported technical roadmap for vision-centric intelligent mental health risk monitoring systems in carceral settings, along with a validated multimodal signal fusion architecture.

(1)The paper presents an implementation of an active, objective, and non-contact psychometric monitoring paradigm, in which the deployment of HMDF-Net changes the operational focus from subjective, reactive in-person assessments to continuous non-contact multimodal behavioral signal acquisition (68), and facial micro-expression analysis is clearly established as the primary link in early-risk identification.

(2) Promote the development of a technical solution that incorporates interpretability mechanisms, and explicitly integrate visual analytics tools such as Gradient-weighted Class Activation Mapping[Grad-CAM] (69) to make artificial intelligence decision pathways visually interpretable for correctional officers. Because of the resulting transparency, operator confidence in algorithmic outputs is greatly increased, which in turn naturally facilitates the design of human-in-the-loop collaborative decision-making architectures (70, 71). Thus, the implementation and deployment of the system can be accelerated.

(3)Help to develop a people-centered closed-loop intervention system by utilizing the HMDF-Net derived technical roadmap and relevant empirical evidence to promote the adoption of non-invasive monitoring technologies in correctional institutions, thus avoiding iatrogenic stigmatization and psychological resistance from intrusive methods, and achieving a true ethical-efficacy balance. Since the approach explicitly values inmate welfare enhancement and dignity preservation, it is natural and logical to design a monitoring-alert-intervention framework that integrates technological deployment with rehabilitative infrastructure, and to use multimodal analytics to deploy wearable biofeedback devices (e. g., heart rate variability monitors) and digital therapeutics, thereby creating a true integrated closed-loop system. Empirical implementations of this framework include real-time biofeedback training (72), application-based mindfulness/affect regulation training (73), and early-warning collaborative intervention systems.

### 4.4 Social value in the field of mental health management

This article undertakes an in-depth, multi-dimensional analysis from three core perspectives: public safety, public health, and social governance.

(1) From the perspective of public safety and judicial governance, HMDF-Net provides quantifiable and traceable scientific means for the mental health management of correctional facilities, which helps to promote global digital rule-of-law governance (74). The traditional mental health risk management model in correctional facilities mainly focuses on passive monitoring, while HMDF-Net may help facilitate a potential shift from “post-event handling” to “pre-event prevention”. Because continuous multimodal analytics (facial micro-expressions, prosodic dynamics, semantic constructs) lend themselves naturally to dynamic neurobehavioral tracking, they are extremely useful for early, reliable detection of suicide/self-harm propensities and aggression trajectories, hence enabling proactive interventions that mitigate institutional security incidents, reduce recidivism, and ultimately promote societal stability (6).With the continuous development of generative artificial intelligence technology, the correctional governance model of human-machine collaboration will further optimize the efficiency of resource allocation and promote the upgrading of correctional management towards intelligence and refinement (75).

(2) From the perspective of public health management, HMDF-Net can serve as a potential and efficient pre-triage tool, which may help alleviate the long-term shortage of mental health management and service resources in correctional facilities. More importantly, the implementation of a tiered clinical pathway combining machine-assisted prescreening with specialist validation achieves both objectives: Firstly, it can provide more extensive and precise mental health services for the special group of inmates, effectively address the issue of insufficient coverage in traditional service models, and promote the fairness and accessibility of public health services in the field of correction. Secondly, more importantly, multimodal analytics facilitate evidence-based correctional programming, thus naturally leading to a closed-loop framework (monitoring alerting intervention evaluation) that has been validated in carceral settings (76).

(3) From the perspective of social governance and legal civilization, HMDF-Net represents the cutting-edge development direction of modern psychological correctional management (77). It promotes the transformation of correctional management from experience-driven to data-driven through standardized evaluation processes and intelligent analysis methods, achieving refinement and transparency in the management process. Meanwhile, the application of this framework technology also reflects the core concept of humanitarian justice: on the premise of ensuring institutional security, it pays more attention to the mental health rights and personal dignity of inmates, achieving a balance between security guarantees and rights protection. By constructing a replicable and sustainable technical application framework, the multi-modal evaluation technology not only strengthens judicial authority but also promotes the improvement of public welfare (78).

### 4.5 Limitations and future work

Despite achieving promising results on a specific dataset, this study has several limitations that point the way for future work:

The robustness of the model under real-world interference needs further verification. The current research uses data collected in a standardized and quiet interview room. The pre-processing steps include audio denoising and removing low-quality or occluded visual frames. Therefore, the reported performance cannot directly prove that the model is robust against severe environmental challenges, such as high-level background noise (e.g., from adjacent conversations or running equipment), severe facial occlusion (e.g., due to camera placement limitations), or complete modality loss (e.g., audio device failure). Although the proposed dynamic fusion mechanism can handle changes in modality quality in its architectural design, its effectiveness under such extreme conditions still needs empirical verification. We have identified the following key steps for future verification: (1) Controlled interference experiments: Systematically evaluate the model’s performance under synthetic interferences of different severities (e.g., additive noise at multiple signal-to-noise ratio levels, random frame masks with different occlusion ratios, simulated modality loss). (2) Field data collection: Collect multimodal data in ecologically valid environments, such as actual prison cells, visitation rooms, or workshop areas, where there are natural environmental interferences. (3) Cross-condition assessment: Compare the model’s performance under standardized and naturally recorded conditions to quantify the performance gap and determine failure modes.

The held-out internal subset (n=8) is too small to serve as external validation, nor does it support meaningful conclusions about real-world generalization. Our sample—76 inmates from three Eastern China correctional facilities—offers limited demographic diversity, and the absence of cross-institution leave-one-out validation further restricts any claims about cross-regional transferability. Ongoing work is therefore building multi-site datasets to enable standardized algorithm verification across diverse detention settings.

Performance also drops markedly when inmates deliberately suppress emotional expression or present blunted affect, since our framework relies exclusively on external audiovisual and textual behavioral cues. Integrating physiological indicators resistant to volitional control—heart rate variability, for instance—should improve both robustness and interpretability, as multimodal physiological features have been shown to enhance detection model reliability. Additionally, the current static classification design does not capture longitudinal dynamics across depressive onset, progression, or remission. Future longitudinal observations paired with causal inference methods will seek early warning biomarkers and trajectory patterns specific to this population.

On the practical side, the contactless video acquisition pipeline introduces inherent privacy risks. Our deployment protocol mandates comprehensive data desensitization, strict access controls, and independent oversight. Ethically, all model outputs are constrained to auxiliary early-screening alerts and never substitute for formal psychological assessment, consistent with WHO Digital Health Guidelines (2023). Regular algorithm bias audits per ISO/IEC 24028:2020 will also be conducted to prevent misclassification and avoid unnecessary stigmatization.

## 5 Conclusion

HMDF-Net delivers a scalable non-invasive screening protocol for correctional mental health with preliminary empirical validation and operational efficiency, alongside a cross-modal affective computing pipeline adapted to resource-limited contexts. Experimental results suggest domain-specific barriers—sparse multimodal data and low signal-to-noise ratios—may impose greater performance restrictions than computational resource scale. Follow-up work will prioritize multi-center verification and physiological signal fusion with longitudinal modeling, aiming to potentially build a more comprehensive digital screening framework for correctional mental health assessment.

## Data Availability

The original contributions presented in the study are included in the article/**Supplementary Material**. Raw multimodal data (video, audio, and transcripts) cannot be made publicly available because of privacy and ethical restrictions governing data collected in correctional facilities; de-identified aggregated data and analysis code are available from the corresponding author on reasonable request.
