# Supplementary material for: HMDF-Net: a transfer learning-based heterogeneous multimodal dynamic fusion network for depression detection among inmates in correctional facilities
Source: Front Psychiatry. 2026 Sep 1;17:1861633. doi: 10.3389/fpsyt.2026.1861633 (PMC13576147; doi:10.3389/fpsyt.2026.1861633)
Supplement: Supplementary file 1 [file DataSheet1.pdf]

Supplementary Material

1 Supplementary Figures and Tables

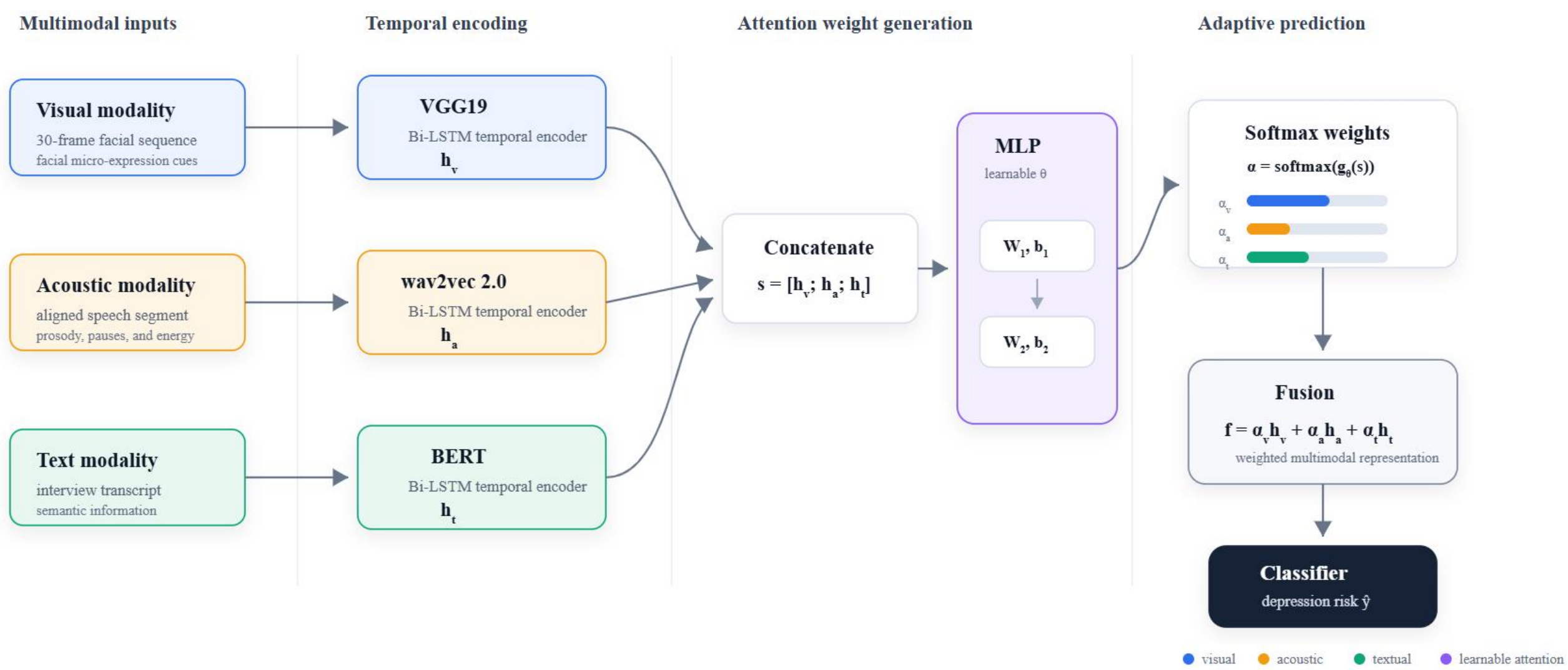

Supplementary Figure 1. Detailed workflow of the Dynamic Attention Fusion Module, which generates time-adaptive modality weights through a learnable parameterised attention network to realize adaptive multimodal fusion.

Supplementary Table 1. Participant and multimodal segment distribution across five cross-validation folds and the held-out internal validation set

Fold	Training Participants	Training (Depressed / Non-depressed)	Training Segments	Validation Participants	Validation (Depressed / Non-depressed)	Validation Segments
Fold 1	54	12 / 42	864	14	4/10	224
Fold 2	54	13 / 41	864	14	3/11	224
Fold 3	54	13 / 41	864	14	3/11	224
Fold 4	55	13 / 42	880	13	3/10	208
Fold 5	55	13 / 42	880	13	3/10	208
Held-out Validation	—	—	—	8	3/5	128

Note. Each participant generates 16 multimodal interview segments. Although model training and inference are conducted at the segment level, all data splits are grouped by participant ID. All original clips and augmented samples from one participant are assigned to the same fold to eliminate cross-fold information leakage.

Supplementary Table 2. Evaluation Protocol for LLMs

Supplementary Table S2 Evaluation Protocol for LLMs

Item	Configuration
Compared Models	Qwen2.5-7B-Instruct；GLM-4-9B
Input Format	Textualized visual, auditory, and transcribed text summaries
Few-shot Example Count	6 examples
Example Composition	3 depressed cases, 3 non-depressed cases
Source of Examples	Current training fold, no overlap with test participants
Decoding Parameters	temperature = 0；top_p = 1.0；max_new_tokens = 16
Output Labels	Depressed / Non-depressed
Class Mapping	Positive label assigned to depressed cases; negative label assigned to non-depressed cases
Handling of Invalid Outputs	Retry once with format constraint; if still invalid, mark as misclassification

Supplementary Table 3. Probability Stratification Results of the Development Set

Probability Interval	Risk Level	Non-Depressed Segments	Depressed Segments	Total Number of Segments in this Interval	Proportion	Actual Depression Rate
0.0-0.2	None/Extremely Mild	427	6	433	39.8%	1.4%
0.2-0.4	Mild	265	22	287	26.4%	7.7%
0.4-0.6	Moderate	93	42	135	12.4%	31.1%
0.6-0.8	Severe	40	110	150	13.8%	73.3%
0.8-1.0	Extremely Severe /Emergency	7	76	83	7.6%	91.6%
Total	-	832	256	1088	100%	23.5%

Supplementary Table 4. Probability Stratification Results of the Held-out Validation Set

Probability Interval	Risk Level	Non-Depressed Segments	Depressed Segments	Total Number of Segments in this Interval	Actual Depression Rate
0.0-0.2	None/Extremely Mild	35	0	35	0.0%
0.2-0.4	Mild	25	0	25	0.0%
0.4-0.6	Moderate	10	3	13	23.1%
0.6-0.8	Severe	8	27	35	77.1%
0.8-1.0	Extremely Severe /Emergency	2	18	20	90.0%
Total	-	80	48	128	37.5%

Supplementary Table 5. Computational efficiency metrics of the full multimodal screening pipeline

Module	Parameters	FLOPs	Peak Memory / VRAM	Latency per Segment	Throughput
Face Detection and Alignment (OpenFace)	—	Not measured	~0.8 GB CPU RAM	0.78 s	1.28 segments/s
Visual Feature Extraction (VGG19, 30 frames)	143.7 M	~0.59 TFLOPs	~2.1 GB GPU VRAM	0.095 s	10.5 segments/s
Audio Denoising, Resampling and Alignment	—	Not measured	~0.3 GB CPU RAM	0.035 s	28.6 segments/s
Voice Feature Extraction (wav2vec 2.0）	95.0 M	~0.08 TFLOPs	~1.4 GB GPU VRAM	0.135 s	7.4 segments/s
Text Transcription / ASR (Whisper-tiny)	39.0 M	~0.04 TFLOPs	~1.1 GB GPU VRAM	0.310 s	3.2 segments/s
Text Encoding (BERT, 128 tokens)	102.3 M	~0.02 TFLOPs	~0.9 GB GPU VRAM	0.012 s	83.3 segments/s
Bi-LSTM plus Dynamic Attention Fusion	6.8 M	~0.001 TFLOPs	~0.4 GB GPU VRAM	0.005 s	200.0 segments/s
End-to-End Pipeline	~386.8 M	~0.73 TFLOPs	~5.2 GB GPU VRAM	~1.37 s	~0.73 segments/s

2 Supplementary Prompt Templates

```
You are assisting with depression-risk screening in a correctional mental-health study.
Classify the participant into one of the following two labels:
Depressed
Non-depressed
Use only the provided evidence. Do not invent clinical facts.
Return only one label.
Few-shot examples:
[Example 1]
Visual summary: ...
Acoustic summary: ...
Transcript: ...
Label: Depressed
[Example 2]
Visual summary: ...
Acoustic summary: ...
Transcript: ...
Label: Non-depressed
...
Test participant:
Visual summary: {visual_summary}
Acoustic summary: {acoustic_summary}
Transcript: {transcript}
Answer only one label:
```
