# Supplementary material for: HMDF-Net: a transfer learning-based heterogeneous multimodal dynamic fusion network for depression detection among inmates in correctional facilities
Source: Front Psychiatry. 2026 Sep 1;17:1861633. doi: 10.3389/fpsyt.2026.1861633 (PMC13576147; doi:10.3389/fpsyt.2026.1861633)
Supplement: Supplementary file 2 [file DataSheet2.pdf]

You are assisting with depression-risk screening in a correctional mental-health study.

Classify the participant into one of the following two labels:

Depressed

Non-depressed

Use only the provided evidence. Do not invent clinical facts.

Return only one label.

Few-shot examples:

[Example 1]

Visual summary: ...

Acoustic summary: ...

Transcript: ...

Label: Depressed

[Example 2]

Visual summary: ...

Acoustic summary: ...

Transcript: ...

Label: Non-depressed

...

Test participant:

Visual summary: {visual_summary}

Acoustic summary: {acoustic_summary}

Transcript: {transcript}

Answer only one label:
